# Identification and Validation of a Mitochondria Calcium Uptake-Related Gene Signature for Predicting Prognosis in COAD

**DOI:** 10.7150/jca.81811

**Published:** 2023-03-21

**Authors:** Jianjun Zhu, Wentao Zhang, Jingjia Chang, Jin Wu, Hao Wu, Xintong Zhang, Zhigao Ou, Ting Tang, Li Li, Ming Liu, Yongfan Xin

**Affiliations:** 1 Department of Cell Biology and Medical Genetics, Shanxi Medical University, Department of Hepatological Surgery, First Hospital of Shanxi Medical University, Taiyuan, Shanxi, China.; 2 Department of Cell Biology and Medical Genetics, Shanxi Medical University, Taiyuan, Shanxi, China.; 3 Department of Molecular & Cellular Biology, Roswell Park Comprehensive Cancer Center, Elm and Carlton Streets, Buffalo, NY 14263, USA.; 4 Department of oncology and vascular intervention, First Hospital of Shanxi Medical University, Taiyuan, Shanxi, China.

**Keywords:** colon adenocarcinoma, gene signature, overall survival, prognosis, immunetherapy, mitochondrial calcium uniporter

## Abstract

**Background:** Mitochondrial calcium uniporter (MCU) complex has been reported to be associated with the tumor occurrence and development in varieties of malignancies. However, the role of MCU complex in colon adenocarcinoma (COAD) remains unclear. Therefore, we constructed a risk score signature based on the MCU complex members to predict the prognosis and response to immunotherapy for patients with COAD.

**Methods:** The MCU complex-associated risk signature (MCUrisk) was constructed based on the expressions of MCU, MCUb, MCUR1, SMDT1, MICU1, MICU2, and MICU3 in COAD. The immune score, stromal score, tumor purity and estimate score were calculated by the ESTIMATE algorithm. We systematically evaluated the relationship among the MCUrisk, mutation signature, immune cell infiltration, and immune checkpoint molecules. The response to immunotherapy was quantified by the Tumor Immune Dysfunction and Exclusion (TIDE).

**Results:** Our results showed that high score of MCUrisk was a worse factor for overall survival (OS) in COAD, and MCUrisk score was significantly higher in advanced COAD. The mutation landscape was different between the MCUrisk-high and MCUrisk-low groups, and the mutation rate of TP53 was remarkably higher in MCUrisk-high group, which strongly suggested TP53 mutation might be associated with mitochondrial calcium dyshomeostasis in COAD. Furthermore, MCUrisk score was negatively correlated with tumor mutation burden (TMB), and combining risk score and TMB as a novel index was better than TMB alone in predicting the prognosis for COAD patients. The compositions of Tregs and M0/M2 macrophages were significantly increased in MCUrisk-high group, whereas CD4^+^ T cells was significantly decreased in MCUrisk-high group. Consistently, the immune score was lower in MCUrisk-high group. The expression levels of immune checkpoint molecules were negatively correlated with the MCUrisk score, including CD58 and CD226. Furthermore, a lower MCUrisk score indicated better response to immunotherapy, and combining risk score and immune score was a novel indicator to precisely predict the response to immuotherapy for COAD patients.

**Conclusion:** Altogether, a novel MCUrisk signature was constructed based on the mitochondrial calcium uptake-associated genes, and a lower MCUrisk score may predict better OS outcome and better response to immunotherapy in COAD.

## Introduction

Colon adenocarcinoma (COAD) is the global leading cause of cancer-associated death 1. Despite the development in the prevention and treatment of COAD, the 5-year overall survival (OS) rate of COAD remains less than 20% 1. Until now, the pathogenesis of COAD is still unclear, and the tumor heterogeneity impeded the precise prediction for individual patients' prognosis in COAD 1, 2. Therefore, more accurate and individual evaluation for patients with COAD remain a great challenge.

Mitochondrial Ca^2+^ dyshomeostasis have been associated with different pathological conditions, including cancer 3. Alterations in calcium flux can affect the functions of mitochondria, and increased evidence indicated that the mitochondrial calcium-handling machinery and mitochondrial calcium homeostasis were altered in kinds of malignancies 3. The Ca^2+^ derived from the extracellular environment or released by the intracellular stores passes across the outer mitochondrial membrane through the voltage-dependent anion channel (VDAC) and reaches the mitochondrial matrix by the mitochondrial calcium uniporter (MCU) complex, located at the inner mitochondrial membrane 3. MCU complex is composed of MCU, MCUb, MCUR1, MICU1, MICU2, MICU3, and SMDT1 4. Altering the expressions or activities of MCU complex members have been linked to tumor development 5-7. Numerous evidences in malignancy demonstrated that MCU complex members influenced patients' prognosis 8, 9. For instance, MCU was significantly up-regulated in CRC tissues, and up-regulated MCU was associated with poorer prognosis in patients with CRC 6. MCUR1 and MICU1 also influenced patients' prognosis and tumor occurrence and progression. MCUR1 expression was significantly increased in HCC with metastasis and associated with tumor progression 10, 11. MICU1 suppressed mitochondrial calcium influx, and elevated MICU1 expression was observed in many kinds of malignancies, which was associated with poor clinical outcomes 12. Furthermore, the abnormal expressions and activities of MCU complex members also could be used as biomarkers to predict whether patients could benefit from therapy, including chemotherapy and immunotheray 13, 14. Given the vital functions and the inconsistent reports of individual MCU complex members in the initiation and progression of malignancies, constructing a prognostic model based on MCU complex members might be an effective strategy to precisely predict the prognosis and benefits from immunotherapy in COAD.

In the present study, based on the public RNA-seq data from The Cancer Genome Atlas (TCGA) dataset and gene expression omnibus (GEO) datasets, we constructed a MCU complex-associated risk signature (MCUrisk) to predict prognosis and responses to immunotherapy in patients with COAD. Furthermore, based on the MCUrisk model, we systematically explored the relationships between the risk score and mutation landscape, tumor mutation burden (TMB), immune cell infiltration, and immune checkpoint molecules, respectively.

## Materials and Methods

### Colon adenocarcinoma data from TCGA and GEO database

In the present study, the transcriptional expression data of COAD was downloaded from TCGA and GEO databases, and the expressions of MCU complex members were analyzed in tumor tissues and normal tissues. The clinical information of the COAD samples was presented in Table 1. Detail information of the MCU complex members were presented in Supplemental Table 1. In the present study, TCGA-COAD was used as training dataset, and GSE17536, GSE29623 and GSE39582 were used as validation datasets 15-17. The inclusion criteria were: 1) patients were pathologically diagnosed as COAD; 2) the patients have complete clinicopathological information. The exclusion criteria were: 1) patients with co-existing cancers of other tissues; 2) patients whose COAD samples lacked RNA-sequencing data; 3) patients who lack survival time and survival status; and 4) the follow-up with 0 day, resulting in the enrolling of a total of 430 patients (Table 1).

### Clinical-Pathological Analysis

The pathological characteristics of patients with COAD were downloaded from TCGA (https://gdc.cancer.gov/). All data are free online, and does not require patient consent or other permissions. The use of the data does not violate the rights of any person or any institution. The numeric values were split at the median and compared between high-risk and low-risk groups. Pearson's chi-square (χ2) test was used to compare these sets of categorical variables.

### Survival analysis in COAD patients

The association between the expressions of MCU complex members and overall survival (OS) and disease-free survival (DFS) of COAD patients were analyzed by Kaplan-Meier Plotter 18. Patients were divided into high-expression and low-expression groups according to the median expression value of each gene. The correlation between the mRNA expression of MCU complex members and the pathological stage of COAD patients was analyzed by GEPIA2 19.

### The prognosis value of the MCUrisk signature

A risk model was constructed based on MCU complex members through R software, TCGA-COAD was used as training dataset, and GSE17536, GSE29623 and GSE39582 were used as validation datasets. Patients in each cohort were divided into high-risk and low-risk groups according to the median value of risk score. The overall survival between subgroups was analyzed by R package ("survival" and "survminer"), and the receiver operating characteristic (ROC) curve was plotted through R package ("survivalROC").

### Gene mutation analysis, protein-protein interaction (PPI) network analysis and functional enrichment analysis

The mutation data of COAD samples were obtained from the cBioPortal 20. The mutation landscape of MCUrisk signature was performed by R packages("maftools"). PPI network was analyzed through STRING database 21, and gene-gene interaction network was analyzed through GeneMANIA 22. The functional enrichment analysis of MCU and its co-expression genes were analyzed by R package ("clusterProfiler", "ggplot2", "enrichplot").

### Immune cell infiltration and responsiveness to immunotherapy

The immune checkpoint molecules list was downloaded from the literature 23. We analyzed the correlation between the expressions of immune checkpoint molecules and the mRNA expressions of MCU complex members in COAD. The immune cell composition between the high-risk and low-risk groups were estimated by CIBESORT 24. The lists of immune cells signatures were downloaded from TISIDB 25. Estimated score, immune score, stromal score and tumor purity were calculated through the ESTIMATE algorithm 26. The TIDE algorithm was used to predict the possibility of response to anti-PD1 and anti-CTLA4 immunotherapy in COAD 27.

### Other statistical analyses

Statistical analyses were performed with GraphPad Prism 8.0 software. Multiple comparisons were performed with the ANOVA test. The correlation between two variables was assessed with Spearman's rank correlation coefficient. * *P* < 0.05, ** *P* < 0.01.

## Results

### Bioinformatic analysis revealed differential expressions of MCU complex members in COAD

**Figure 1:**
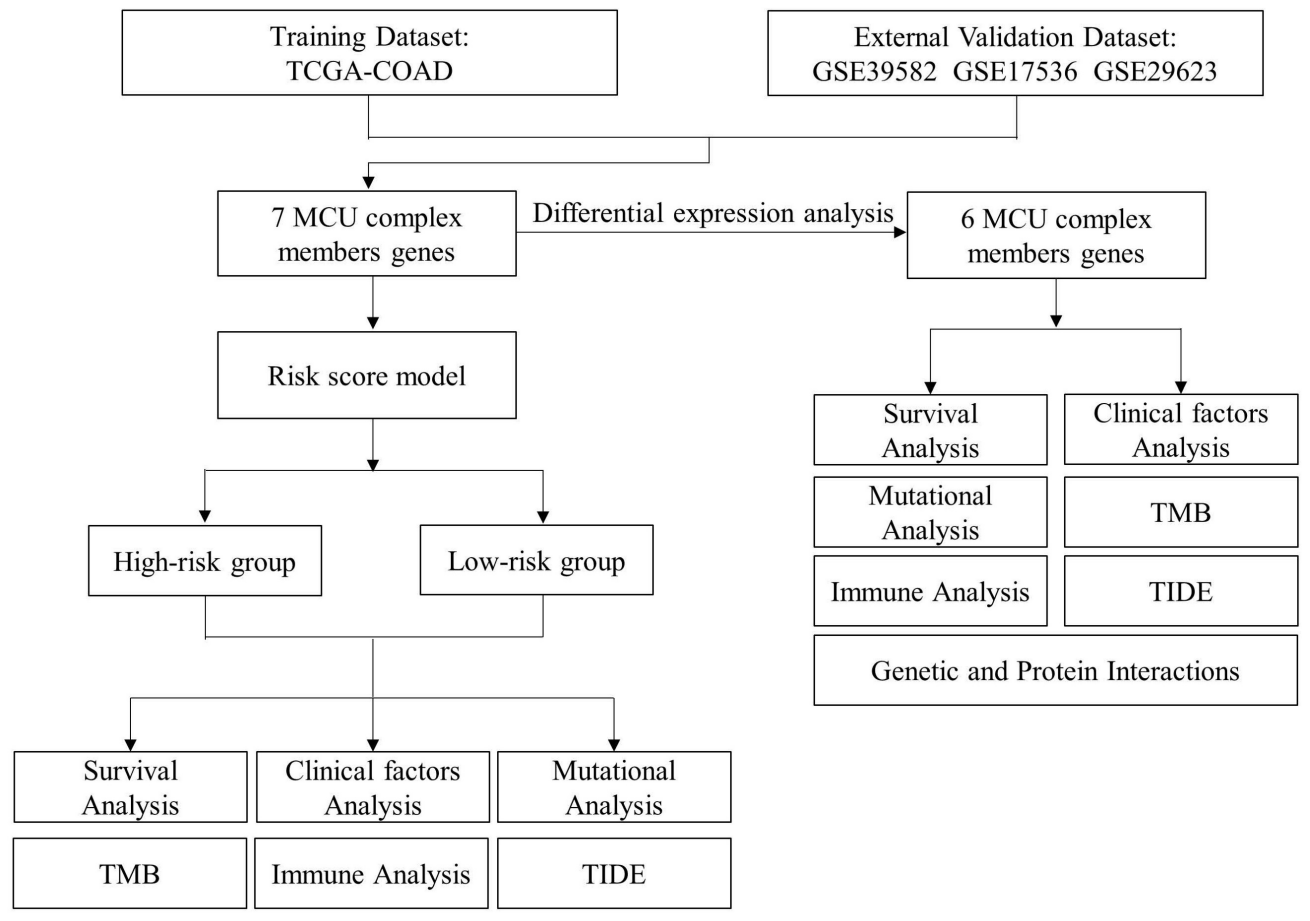
Workflow diagram. The flowchart graph of this study.

**Figure 2:**
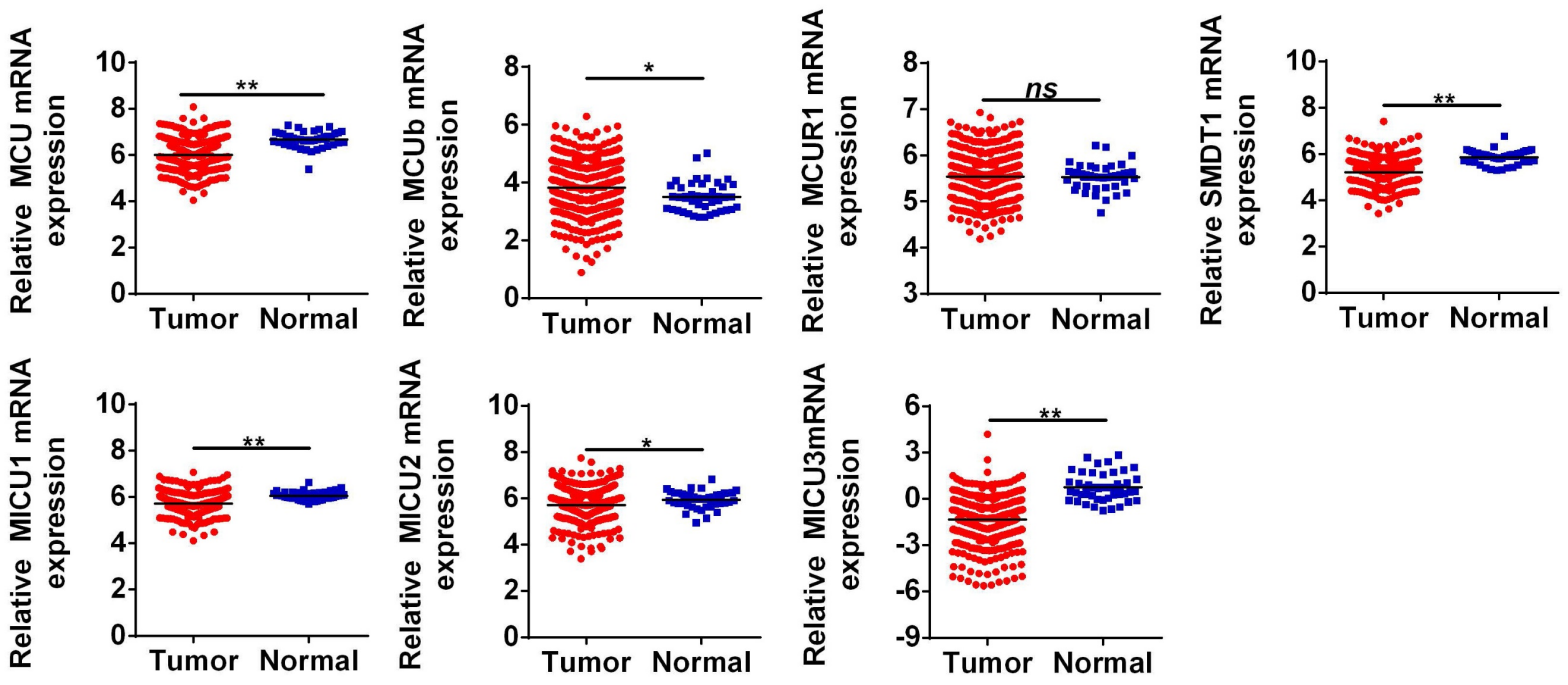
The mRNA expressions of MCU complex members in colon cancer. *P < 0.05; **P < 0.01.

The general process of the present study was presented in **Figure 1**. First, the expression levels of MCU complex members in COAD and normal colon tissues were analyzed using the RNA-Seq data from TCGA. As shown in **Figure 2**, the mRNA expressions of MCU, SMDT1, MICU1, MICU2, and MICU3 were significantly down-regulated, whereas MCUb was significantly up-regulated in COAD, compared to that in normal colon tissues. Consistent with the results from TCGA, the results from GSE39582 datasets showed that the mRNA expressions of MCU, SMDT1, and MICU2 were significantly down-regulated, whereas MCUb was significantly up-regulated in COAD (**Figure S1**).

**Figure 3:**
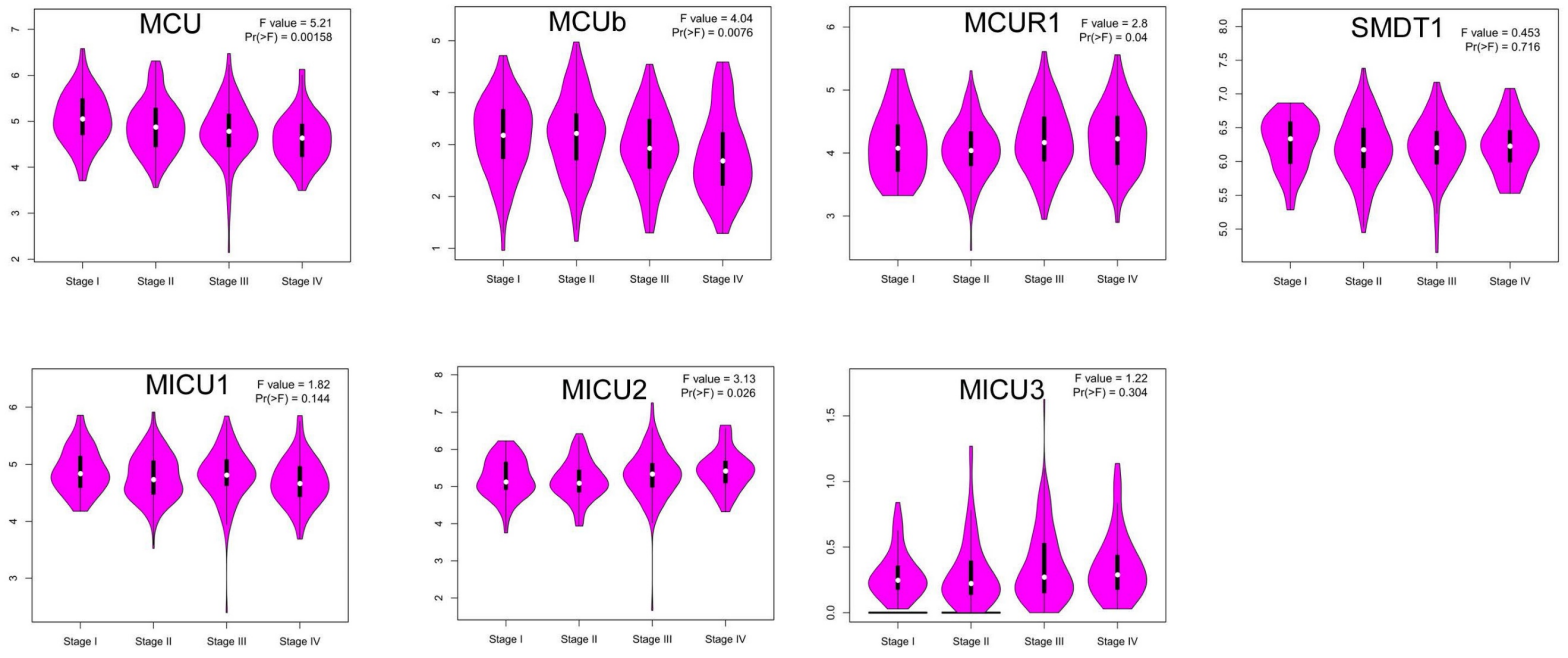
Correlation between the mRNA expression of MCU complex members and the pathological stage of patients.

Then, we explored whether the abnormal expressions of MCU complex members were associated with pathological stage in COAD. The results showed that the expression of MCU and MCUb gradually decreased with the pathological progression of COAD, whereas the expression of MICU2 gradually increased with the pathological progression of COAD (**Figure 3**). Furthermore, we systematically analyzed the relationships between the expressions of MCU complex members and other clinicopathological characteristics in COAD. Our results showed that the expressions of all MCU complex members were not associated with age, gender, and T stage. However, the decreased expressions of MCU and MCUb was associated with the advanced N and M stage. In addition, the decreased expression of MICU1 and the increased expression of MICU2 were associated with the advanced M stage (**Figure S2**). Taken together, these results indicated that MCU complex members, especially MCU, MCUb, and MICU2, might functioned important roles in the pathological progression of COAD.

### The Prognostic value of MCU complex members in COAD

**Figure 4:**
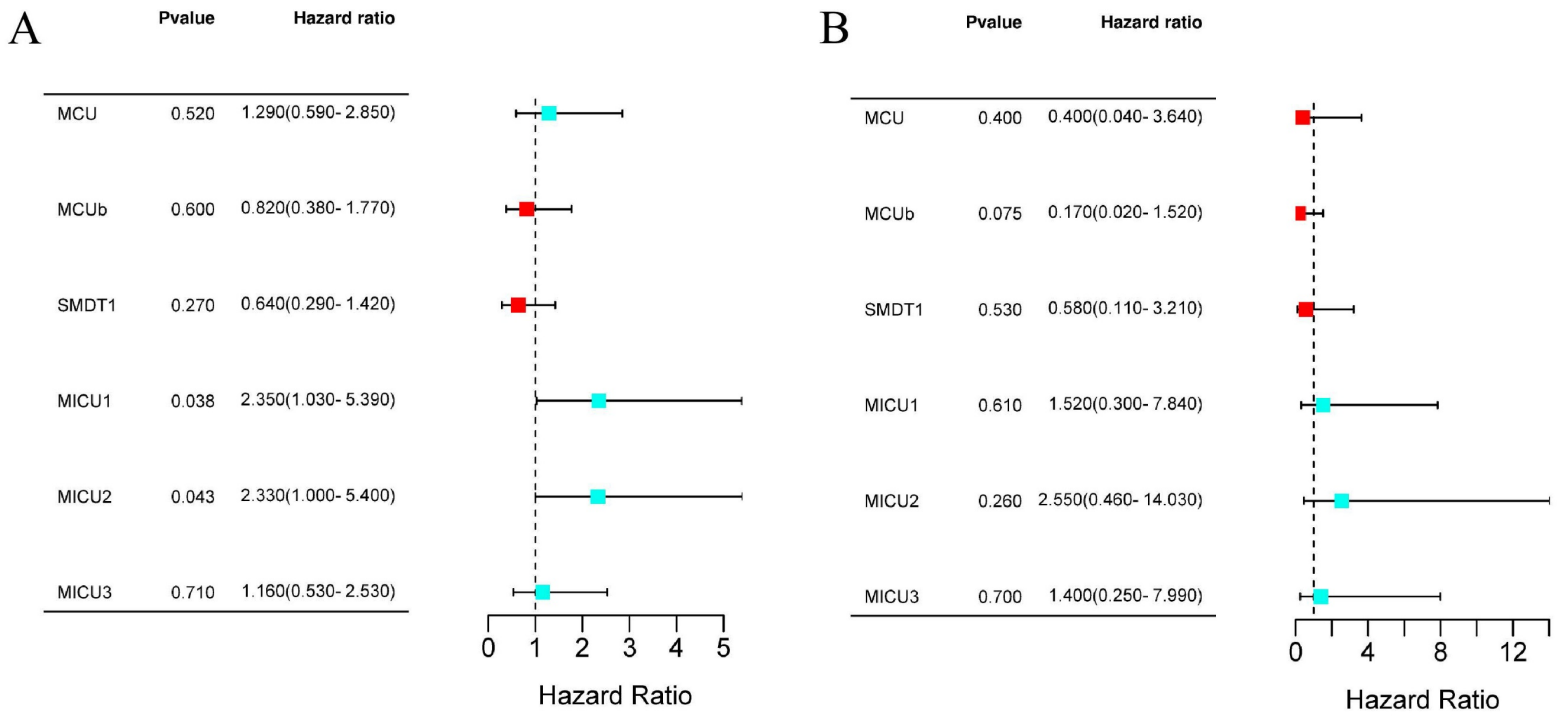
The prognostic value of individual MCU complex members in COAD. **a**, overall survival (OS) analyses. **b**, disease-free survival (DFS) analyses.

Next, we evaluated the prognostic values of MCU complex members in COAD. The results from Kaplan-Meier Plotter database showed that patients with high expression of MICU1 or MICU2 had a shorter OS (**Figure 4A and Figure S3A**). Moreover, the expression of all MCU complex members was not associated with the disease-free survival (DFS) (**Figure 4B and Figure S3B**).

### Gene mutation, protein-protein interaction network and correlation analyses of MCU complex members in COAD

Gene mutation was a causative factor in the process of initiation and progression in malignancies. Given to that, we firstly analyzed the genetic alternations of the MCU complex members by cBioPortal. As shown in **Figure S4A**, the mutation rates of MCU, MCUb, MCUR1, MICU1, MICU2, and MICU3 were 2.3%, 0.5%, 0.9%, 1.4%, 5.0%, and 8.0% in COAD samples, respectively. No genetic alternation of SMDT1 was observed in COAD samples. As tumor mutation burden (TMB) was a robust prognostic indicator in COAD, we analyzed the relationship between the expressions of MCU complex members and TMB in COAD. As shown in **Figure S4B**, the expressions of MCUb, MCU, and MICU1 were significantly positively correlated with TMB (all *r*>0.2, *P*<0.01). As APC, TP53, and PIK3CA were the high-frequency mutated genes n COAD, we analyzed the mutated relationship between the MCU complex members and the high-frequency mutated genes in COAD. As shown in **Figure S4C**, MICU2 and APC showed significant mutant exclusion in COAD (*P*<0.05). Taken together, these results indicated that the expressions of MCUb, MCU, and MICU1 were positively correlated with the TMB in COAD, strongly suggested that the abnormal expressions of MCU complex members might be the causative factor for tumor mutation, which needs to be comprehensively studied in the future.

The PPI network showed that MCU directly interacted with the other members, including MCUb, MCUR1, SMDT1, MICU1, MICU2, and MICU3 (**Figure S4D**). In addition, co-expression of MCU complex members was further analyzed. As shown in **Figure S4E**, there was a strong correlation among the expressions of MCU, MCUb, and MICU1. The results from the GeneMANIA indicated that MCU complex members were primarily associated with SLC25A23, CCDC90B, and EIF2B family members, *et al.* (**Figure S4F**).

### The association between the expressions of MCU complex members and immune cell infiltration in COAD

As the infiltration of immune cells in tumor microenvironment (TME) was closely related to the prognosis and responses to immunotherapy in COAD, we then analyzed the relationship between the expressions of MCU complex members and the distribution of immune cell infiltration in COAD. As shown in **Figure S5,** the compositions of M0 macrophages and CD4 memory resting T cells were significantly different in MCU-high group and MCU-low group, SMDT1-high group and SMDT1-low group, MICU2-high group and MICU2-low group in COAD, respectively. Moreover, the compositions of M0, M1 macrophages, and CD8 T cells were significantly different in MCUb-high group and MCUb-low group in COAD. The compositions of M0 macrophages and activated CD4 memory T cells were significantly different in MICU1-high group and MICU1-low group in COAD. The compositions of T cells regulatory (Tregs), M2 macrophages, and activated mast cells were significantly different in MICU3-high group and MICU3-low group in COAD. Altogether, these results demonstrated that the expressions of MCU complex members was associated with immune cells infiltration in COAD.

### The association between the expressions of MCU complex members and immune checkpoint molecules and immunotherapy

As the expressions of MCU complex members were associated with the immune cell infiltration, and the expressions of immune checkpoint molecules, especially PD-1 (programmed cell death 1, also named as PDCD1), PD-L1 (programmed cell death ligand 1, also named as CD274) and CTLA-4 (cytotoxic T-lymphocyte-associated protein 4), were related with the responses to immunotherapy in COAD, we explored the correlationship between the expressions of MCU complex members and the immune checkpoint molecules. As shown in **Figure S6A**, the expression of PD-1 was significantly up-regulated in MCUb-high subgroup, MICU1-high subgroup, MICU3-high subgroup, and MICU2-low subgroup in COAD. In addition, the expression of PD-L1 was significantly up-regulated in MCU-high subgroup, MCUb-high subgroup, MICU1-high subgroup, MICU3-high subgroup, SMDT1-low subgroup, and MICU2-low subgroup in COAD. The expression of CTLA-4 was significantly up-regulated in MCUb-high subgroup, MICU3-high subgroup, SMDT1-low subgroup, and MICU2-low subgroup in COAD. Furthermore, the relationship between the expressions of MCU complex members and 31 immune checkpoint molecules were systematically analyzed, and our results indicated the expressions of MCUb, MICU1, and MICU3 was positively correlated with the majority of immune checkpoint molecules, whereas MICU2 and SMDT1 was negatively correlated with the majority of immune checkpoint molecules (**Figure S7**). Then, we explored the relationship between the expressions of MCU complex members and the response to anti-PD-1 and anti-CTLA-4 immunotherapy. As shown in **Figure S6B**, the response rate to immuotherapy was significantly higher in patients with higher expression of MCU. The similar results were observed in the patients with higher expression MCUb, and in the patients with lower expression of MICU3. Taken together, these results demonstrated that the expressions of MCU complex members might be related with immunotherapy responses in COAD.

### Construction of MCU complex‑related risk (MCU-risk) signature

**Figure 5:**
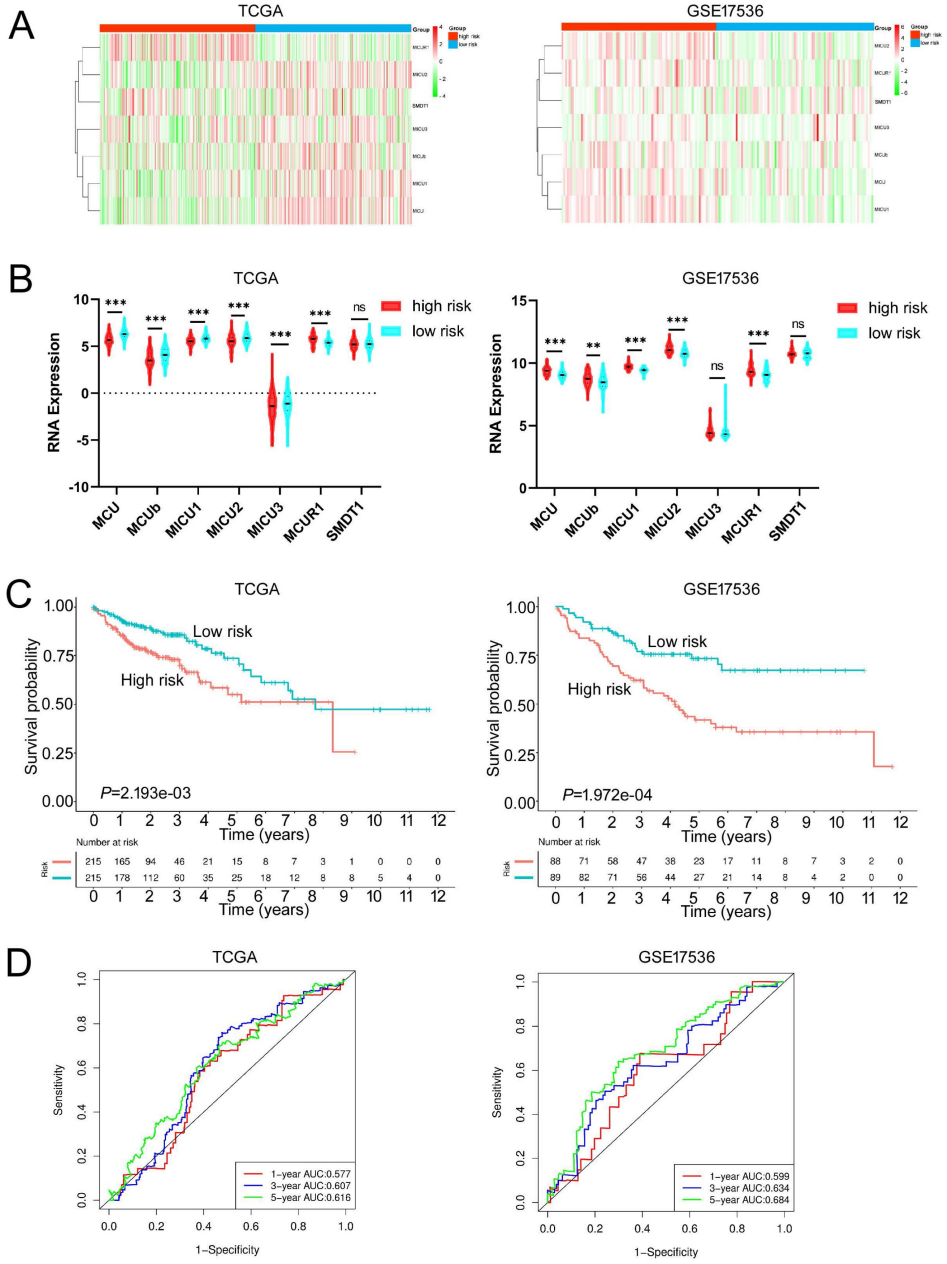
Constructed and validated the MCU complex members-related risk signature. **A** Heatmap of mRNA expression of MCU complex members in low-risk and high-risk group in training dataset (TCGA) and validation dataset (GSE17536). **B** The mRNA expression of MCU complex members in low-risk and high-risk group in training dataset and validation dataset. **C** K-M survival between low-risk and high-risk group in training dataset and validation dataset. **d,** ROC curve and the areas under the curve (AUC) at 1, 3, and 5 years for the risk score in training dataset and validation dataset. **P < 0.01; ***P < 0.001.

Given the MCU complex was composed of MCU, MCUb, MCUR1, SMDT1, MICU1, MICU2, and MICU3, and the individual MCU complex members can't effectively predict the prognosis for patients with COAD, a risk score model was constructed based on the expressions of MCU complex members to evaluate the outcomes of patients with COAD. The TCGA cohort was used as training dataset, and GEO datasets as validation datasets, including GSE39582, GSE17536, and GSE29623. Patients were divided into high-risk group and low-risk group according to the median risk score in each dataset. The results indicated that high-risk group showed a poorer prognosis for patients with COAD both in the training dataset and validation datasets (**Figure 5, and Figure S9**). Furthermore, our results showed that the risk score was significantly higher in advanced COAD tumors (**Figure S8**). Altogether, these results demonstrated that the MCUrisk signature was a novel robust index in predicting the prognosis in patients with COAD.

### The relationship between MCU-risk signature and mutation profile in COAD

**Figure 6:**
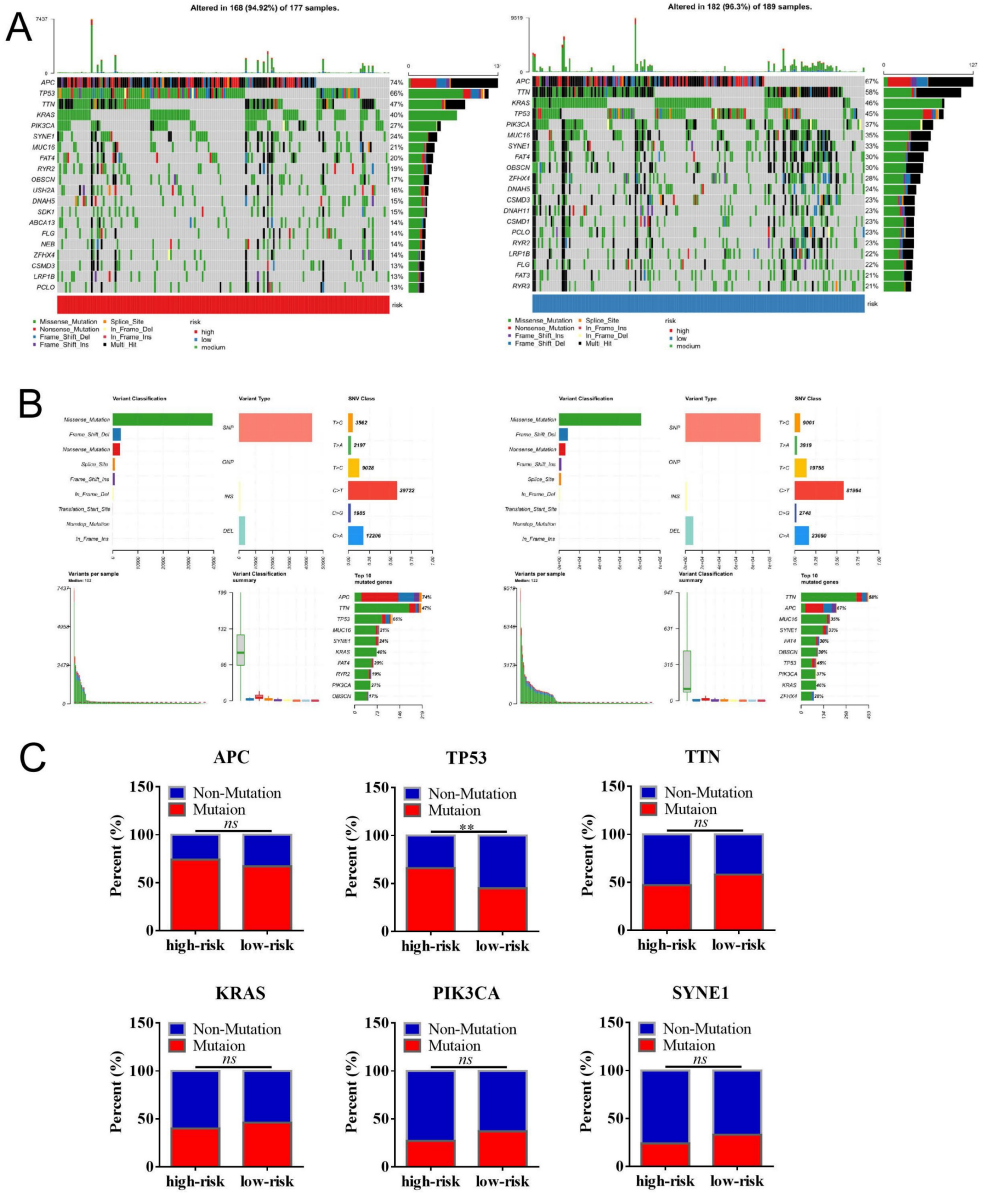
The mutation profile in low-risk and high-risk groups. **a**, Mutation profile of COAD patients in low-risk and high-risk groups. **b**, The summaries of the gene mutation information of risk signature. **c**, The distribution of non-mutation and mutation samples of APC, TP53, TTN, KRAS, PIK3CA, and SYNE1 in the low-risk and high-risk group, respectively.

**Figure 7:**
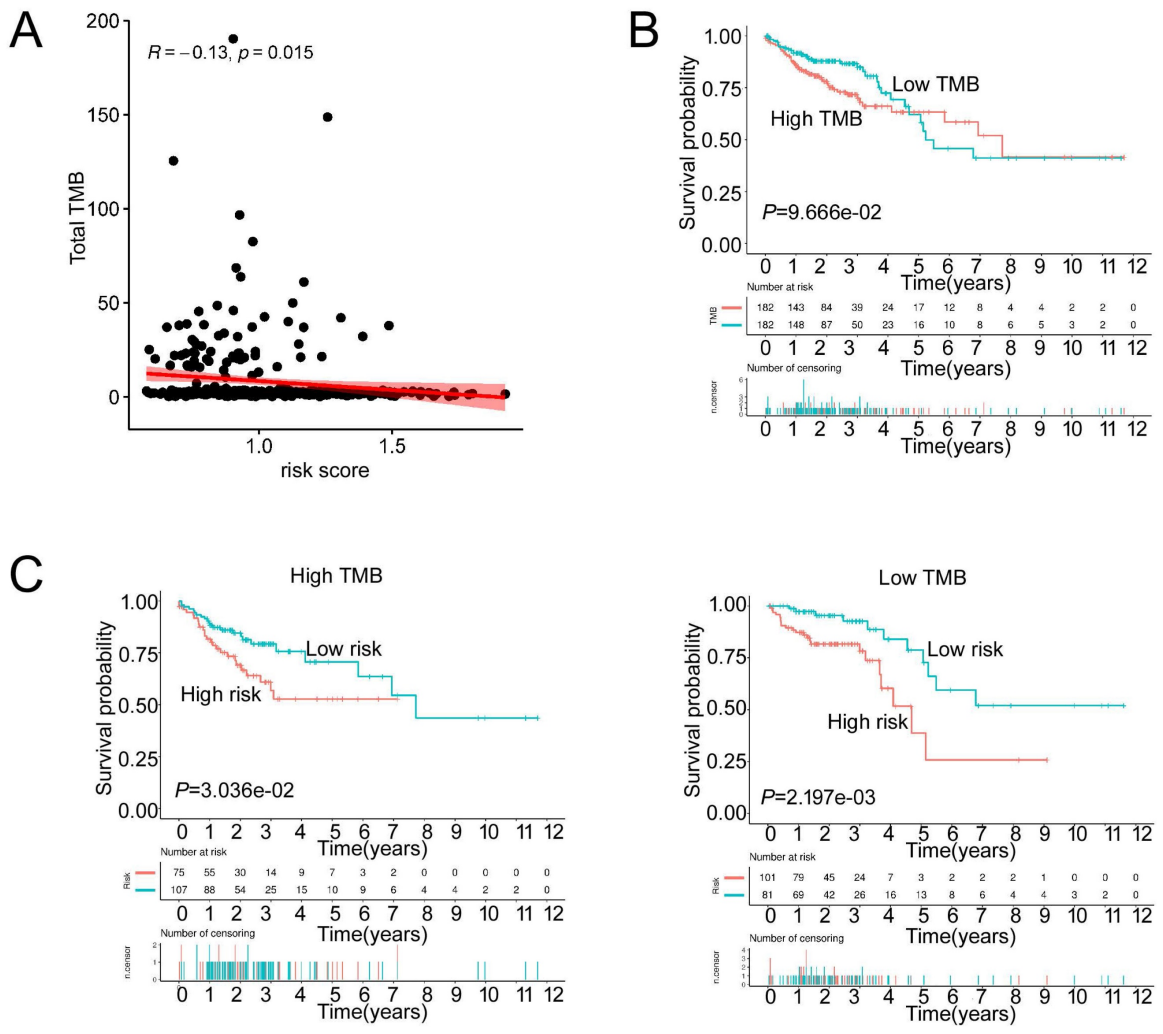
The TMB between low-risk and high-risk groups. **a**, The relationship between the risk score and TMB. **b**, The association of TMB and survival analyses in the training dataset. **c**, K-M survival analyses between low-risk and high risk-group in the high TMB groups and the low TMB groups, respectively.

We then analyzed the genetic alteration to gain more biological insights into the molecular characteristics of MCU-risk using TCGA dataset. Our data showed that APC (74%), TP53 (66%), TTN (47%), KRAS (40%), and PIK3CA (27%) were the top 5 highest mutation genes in the high-risk group, whereas APC (67%), TTN (58%), KRAS (46%), TP53 (45%), and PIK3CA (37%) were the top 5 highest mutation genes in the low-risk group (**Figure 6A**). The genetic alteration information was presented in the bar plot (**Figure 6B**). As shown in **Figure 6C**, the mutation rate of TP53 in the high-risk group was significantly higher compared to the low-risk group, which suggested that high mutation frequencies of TP53 might contribute to the high risk in COAD. We subsequently explored the relationship between the risk score and TMB in COAD. Our results demonstrated showed that the risk score was significantly negatively correlated with TMB (**Figure 7A**). As shown in **Figure 7B**, the high-TMB group had a poorer OS in COAD, but without a statistically significant difference. However, combining risk score and TMB could effectively predict the prognosis in COAD, and our results showed that the patients with high risk score suffered a poorer prognosis compared to the patients with low risk score in low TMB subgroup (**Figure 7C**). The similar results were observed in the high TMB subgroup, which strongly suggested that combining risk score and TMB was a robust prognostic index in COAD.

### Functional enrichment analysis of DEGs between the high-risk and low-risk subgroups in COAD

533 DEGs between the high-risk and low-risk subgroups in COAD were identified and used for functional enrichment analysis by using DVAID 6.8 and Metascape. DEGs between the high-risk and low-risk subgroups were mostly involved in the biological process (BP) including regulation of T cell differentiation in thymus, positive regulation of T cell differentiation in thymus. In terms of molecular functions (MF), these genes were mostly involved in receptor ligand activity, signaling receptor activator activity, and anion transmembrane transporter activity, *et al.* (**Figure S10**). Taken together, these results indicated that MCU complex members were closely related with the function of immune cell in COAD.

### MCU-risk signature predicts the immune cell infiltration and response to immunotherapy in COAD

**Figure 8:**
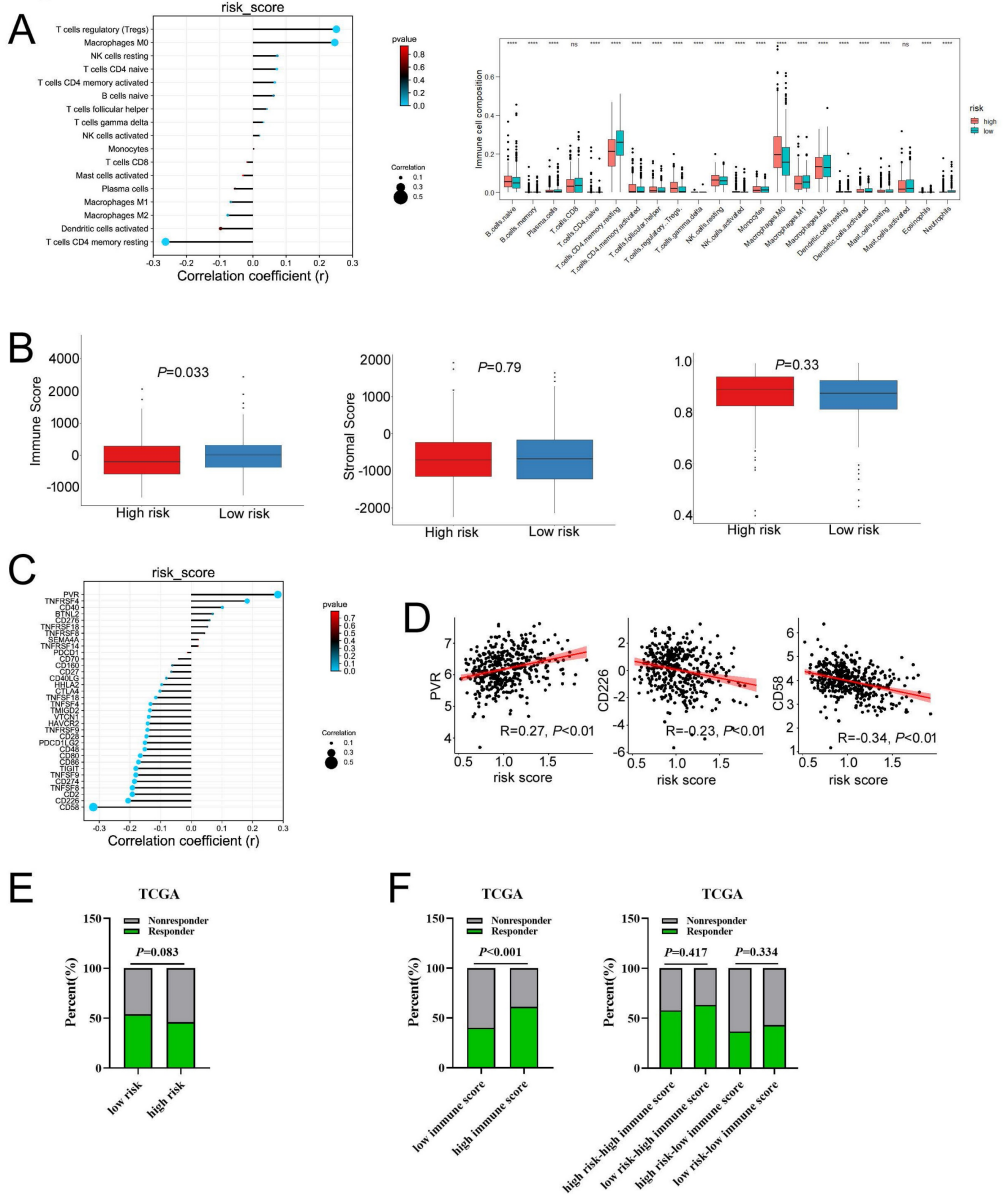
The associations of risk scores of immune cell infiltration and immunotherapy response. **a**, Compared the immune cell fraction between the low-risk and high-risk group. **b**, The correlation between immune, stromal, and tumor purity and risk score in training set. **c**, The relationship between the expression of immune checkpoint molecules and risk score. **c**, The relationship between the expression of PVR, CD226, and CD58 and risk score, respectively. **e**, The distribution of responder and non-responder to anti CTLA-4/PD-1 immunotherapy in the low-risk and high-risk groups and in the low-immune score and high-immune score groups. **e**, The distribution of responder and non-responder to anti CTLA-4/PD-1 immunotherapy with different combinations of risk score and immune score.

To evaluate the relationship between the risk signature and tumor immune microenvironment (TIME), we systematically analyzed the composition of 22 immune cell in the high-and low-risk groups by CIBERSORT algorithm. Our results showed that the proportion of M0 macrophages, M2 macrophages, resting NK cells, and T cells regulatory (Tregs) in the high-risk group were significantly increased, whereas the proportion of M1 macrophages, and CD4 memory resting T cells in the high-risk group were remarkably decreased (**Figure 8A and Figure S11A**). Furthermore, our results showed that the risk score was positively correlated with Tregs and M0 macrophages, whereas negatively correlated with the CD4 memory resting T cells (**Figure 8A**). A correlation analysis was performed to evaluate the relationship between the risk score and the biomarkers of immune cell. The results presented that risk score was negatively correlated with the majority of biomarkers of activated CD4 T cells (**Figure S11B, Figure S12**). Then, the estimated score, immune score, stromal score, and tumor purity were calculated by ESTIMATE algorithm. As shown in **Figure 8B and Figure S11C**, risk score was negatively correlated with immune score in COAD, but not with stromal score, tumor purity, and estimate score. In addition, immune score was significantly lower in the high-risk group compared with that in the low-risk group. Taken together, these results indicated that increased infiltration of Tregs and M0 macrophages-mediated tumor immunosuppressive microenvironment might contribute to the poor survival for patients with COAD.

Then, we explored whether the risk score was related with immune checkpoint molecules in CAOD. The results from correlation analysis showed that the expression of PVR was significantly positively correlated with risk score, whereas CD226 and CD58 were negatively correlated with risk score in COAD (**Figure 8C-D**). Based on the above results, TIDE algorithm was used to predict the possibility of response to anti-PD1 and anti-CTLA4 immunotherapy in the high- and low-risk groups. The result indicated that the rate of respond to anti-PD1 and anti-CTLA4 treatment was higher in low-risk group than that in the high-risk group, but without a statistically significant difference (**Figure 8E**). The rate of immunotherapy responders was significantly higher in high-immune score group (**Figure 8F**). Given to the above results, we evaluated whether combining risk score and an immune score was superior to immune score alone in predicting response to immunotherapy. Interestingly, the results indicated that patients with low-risk score in the low immune score group respond better to anti-PD-1 and anti-CTLA-4 treatment, compared to the patients with high-risk score (**Figure 8F**). Similarly, the consensus results were observed in the high immune score group. Taken together, these results demonstrated that combined with risk score and immune score was a better index in predicting the responses to immunotherapy in COAD.

### Drug Sensitivity Analysis of MCU complex members

**Figure 9:**
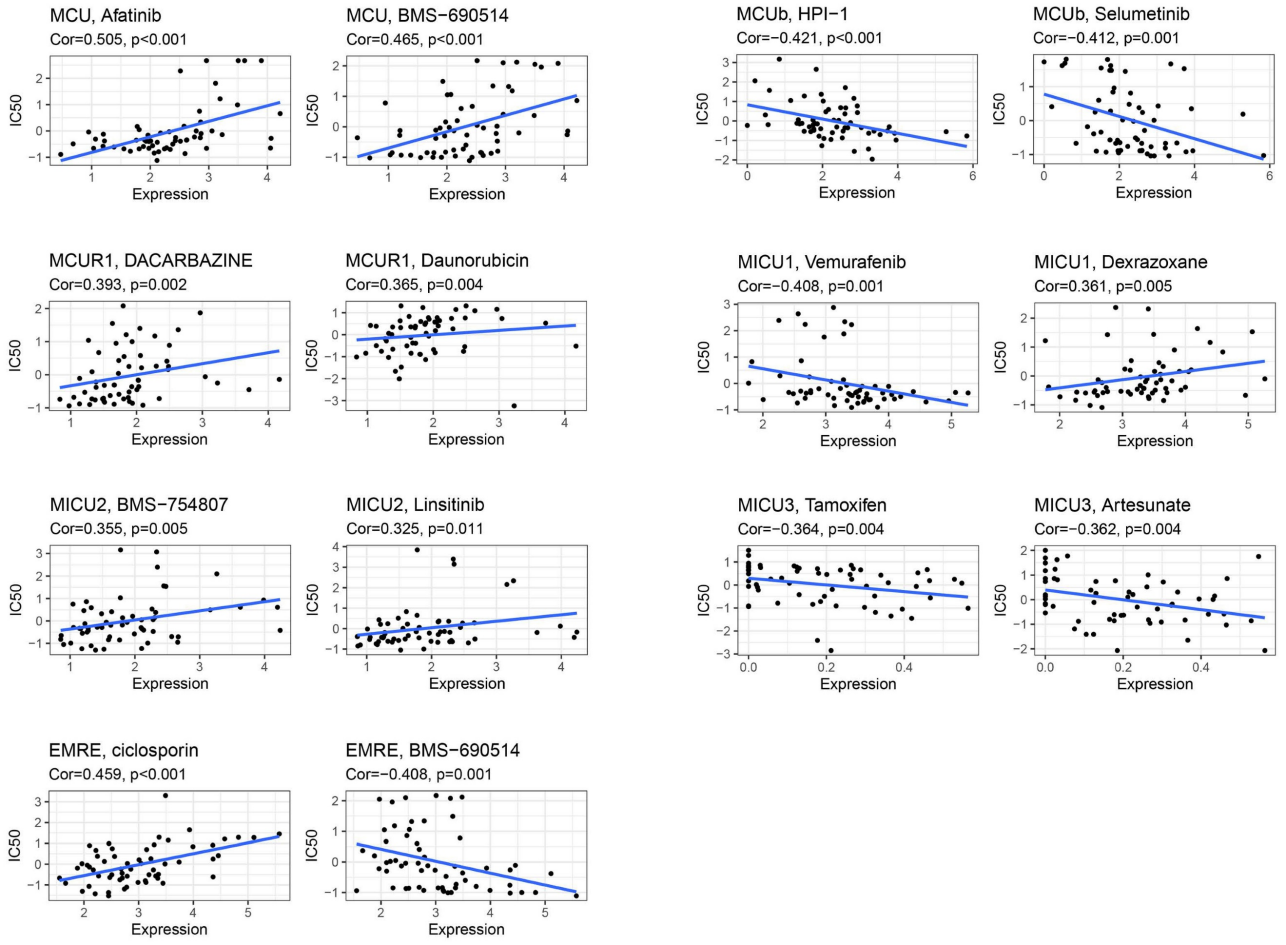
The expression of MCU complex members predicts drug responses of COAD patients.

CellMiner was used to assess the interactions of model genes on drug sensitivity, in order to facilitate better precision treatment of COAD. As shown in** Figure 9**, the top 2 drugs with the highest correlation with the expression of model genes were presented. The patients with low expression of MCU might benefit from Afatinib and BMS-690514, and patients with high expression of MCUb might benefit from HPI-1 and Selumetinib. The patients with low expression of MCUR1 might benefit from Dacarbazine and Daunorubicin, and patients with high expression of MICU1 might benefit from Vemurafenib. The patients with low expression of MICU2 might benefit from BMS-754807 and Linsitinib, and patients with high expression of MICU3 might benefit from Tamoxifen and Artesunate. The patients with low expression of SMDT1 might benefit from ciclosporin.

## Discussion

COAD remains one of the most lethal malignancies in the worldwide with a poor prognosis 1. The incidence of COAD increased in many areas around the world, ranking sixth among all cancer-related death in China 1. Due to the lack of early-set symptoms, the majority of COAD patients were diagnosed at the progressed stage, which led to the poor prognosis 1. Lots of research pointed the pivotal biological roles of mitochondrial Ca^2+^ homeostasis in tumor initiation and development. Mitochondrial uptake calcium mainly through MCU complex in eukaryocyte 3. Recent discoveries identified the uniporter pore-forming subunit MCU and its regulatory molecules, including MCU-dominant negative β subunit (MCUb), essential MCU regulator (EMRE, also named SMDT1), MCU regulator 1 (MCUR1), mitochondrial calcium uptake (MICU) 1, MICU2, and MICU3 3. MCU is the primary mediator of Ca^2+^ influx into mitochondria, which was involved in energy metabolism, ROS production, and programmed cell death 3.

MCU complex members mediated multiple functional roles, which were associated with the carcinogenesis and progression of COAD, including survival, cell proliferation, cell death, metastasis, and chemoresistance 3. In humans, MCU and MICU1 are encoded by a cluster of genes located on chromosome 10q22.1, and MCUb is encoded by the gene located on chromosome 4q25, MCUR1 is encoded by the gene located on chromosome 6q23, MICU2 is encoded by the gene located on chromosome 13q12.11, MICU3 is encoded by the gene located on chromosome 8q22, SMDT1 is encoded by the gene located on chromosome 22q13.2. What is noteworthy is that a strong positive correlation was observed between the expressions of MCU and MICU1, and the cluster analysis further indicated that the expression profiles tendency of MCU and MICU1 were coincident, strongly suggesting that MCU and MICU1 were two co-expressed genes in COAD. Now, MCU complex members were considered as potential therapeutic targets for malignancies treatment, and the agonists or antagonists presented well potential anti-tumor activity in the preclinical assays and clinical trials. For instance, Li *et al.* found that neochlorogenic acid (NA) could bind with MCU, and effectively trigger MCU-mediated calcium overload, followed by leading to mitochondrial dysfunction, and ROS elevation, suggesting NA might be used as small molecule drug for cancer treatment 28. However, the prognostic values and functional roles of MCU complex members in COAD remained elusive.

The abnormal expressions of MCU complex members were reported in a variety of malignancies, including COAD, lung cancer, breast cancer, and hepatocellular carcinoma 6. Liu *et al.* reported that MCU was markedly up-regulated in CRC, and up-regulated MCU was associated with poor prognosis in patients with CRC 6. However, in the present study, the decreased expression of MCU was observed in COAD, and the decreased expression of MCU was not associated with prognosis in patients with COAD. We speculated that different subtype samples enrolled in our study might contribute to the inconsistent results with Liu's study, and more samples should be enrolled to reconfirm the expression and the prognostic value of MCU in COAD. MCUb was highly expressed in high grade gliomas, and the expression of MCUb was inversely correlated with patients' overall survival, indicating that MCUb could be served as a prognostic marker in glioma 29. Consistently, the expression of MCUb was significantly increased in COAD, but the expression of MCUb was not correlated with COAD patients' OS and RFS in the present study. Jin *et al.* reported the expression of MCUR1 was significantly higher in HCC with metastasis and associated with tumor progression 10. Gao* et al.* also reported that MCUR1 was over-expressed in breast cancer, and the high expression of MCUR1 was associated with poor OS and RFS in breast cancer 30. However, Fan *et al.* reported the low expression of MCUR1 was associated with the poor prognosis in ovarian cancer 31. However, inconsistent with the above studies, no significant different expression of MCUR1 was observed in COAD, and the expression of MCUR1 also didn't associated with OS and RFS in COAD. These studies indicated that the expression pattern of MCUR1 might be complicated in different types of malignancies.

Elevated MICU1 expression was characteristic of many cancers, and the high expression of MICU1 was associated with poor clinical outcomes of ovarian cancer 12. However, the results from Li's study showed that low expression of MICU1 indicated poor prognosis in stage I/II and III/IV patients with HCC, respectively 32. Inconsistent with the above studies, the expression of MICU1 was significantly decreased in COAD, but the expression of MCIU1 was not associated with the OS and RFS in COAD. HCC patients with high MCU/MICU2 expression exhibited poor prognosis in OS analysis 32. In the present study, our results showed that high expression of MICU2 was significantly associated with the poorer OS in COAD. Xie *et al.* reported that SMDT1 expression was significantly positive correlated with PDAC prognosis 33. However, in the present study, the expression of SMDT1 was significantly decreased in COAD, but the expression of SMDT1 was not associated with the COAD prognosis. Moreover, our results showed that the expressions of MCU and MCUb decreased as the COAD progressed, whereas the expressions of MCUR1 and MICU2 increased as the COAD progressed.

Although studies have indicated the critical biological role of mitochondrial Ca^2+^ uptake in cancer pathophysiology, MCU complex members in predicting the prognosis of patients with malignancy are incosistent. Given the inaccuracy and inefficiency of the expressions of individual MCU complex members in predicting the prognosis in COAD, a MCU complex-associated risk score (MCUrisk) model was constructed based on the MCU complex members. In the present study, the prognostic value of MCUrisk was evaluated in four independent cohorts, and the COAD patients in the high-risk group suffered poorer OS. As the tumorigenesis and development of COAD were a multi-step processes, and multi-factors and genetic alterations were involved in this process, we systematically explored the genetic alterations of the MCU complex members. Dong *et al.* demonstrated that mutation of MCU Cys-97 presented persistent MCU channel activity with higher [Ca^2+^]_m_ uptake rate, elevated mROS production, and enhanced [Ca^2+^]_m_ overload-induced cell death 34. A homozygous truncating mutation in MICU2 caused abnormal mitochondrial calcium homeostasis and a severe neurodevelopmental disorder 35. Therefore, the genetic alterations of MCU complex members might be the causative factors in occurrence and development of malignancy. Moreover, the mutation landscape was different between the MCUrisk-high and MCUrisk-low groups. What is noteworthy is that the mutation rate of TP53 was remarkably higher in MCUrisk-high group, which strongly suggested TP53 mutation might be associated with mitochondrial calcium dyshomeostasis in COAD. However, the causal relationship between TP53 mutation and mitochondrial calcium dyehomeostasis was unclear, which was worth to study in the further. Furthermore, MCUrisk was negatively correlated with TMB, and combining MCUrisk and TMB as a novel composite index was better than TMB alone in predicting the prognosis for COAD patients.

In the past years, accumulative evidence suggested MCU-dependent mitochondrial Ca^2+^ signaling may regulate immune cell function. For instance, myeloid deletion of MCU mice showed a pronounced decrease in immune cell recruitment in alum-induced peritonitis 13. Yoast *et al.* defined multiple roles of the MCU in regulating lymphocyte activation, and found that MCU knockdown significantly enhanced proliferation of B cells in response to B-cell receptor stimulation 36. Mast cells play a fundamental role in immune system, and activating MCU suppressed mast cell degranulation via increased mitochondrial Ca^2+^ level 37. In the present study, the composition of M0 macrophage was increased, and the composition of CD4 memory resting T cell was decreased in patients with low expression of MCU in COAD. However, the composition rate of mast cell didn't have significant difference between the MCU-high and MCU-low groups. Feno *et al.* reported that MCUb promoted muscle regeneration by controlling macrophage responses 38. Consistent with the above study, our results presented that the compositions of M1 macrophages and CD8^+^ T cells were increased, and the composition of M0 macrophages was decreased in patients with high expression of MCUb in COAD. Except for MCU and MCUb, we also explored the relationship between the infiltration of immune cells and the expression of other MCU complex members, including SMDT1, MICU1, MICU2, and MICU3. The composition of M0 macrophages was increased, and the composition of CD4 memory resting T cells was decreased in patients with low expression of SMDT1 in COAD. The compositions of M0 macrophages and CD4 memory resting T cells were increased in patients with low expression of MICU1 in COAD. The composition of M0 macrophages was increased, and the composition of CD4 memory resting T cells was decreased in patients with low expression of MICU2 in COAD. The compositions of Tregs and mast cells were increased, and the compositions of M0 macrophages and M2 macrophages were decreased in patients with low expression of MICU3 in COAD. Taken together, these results demonstrated that MCU complex members were closely correlated with the infiltration of immune cell, especially including macrophage and CD4^+^ T cells in COAD.

The function of the DEGs between the MCUrisk-high and MCUrisk-low group was explored with GO and KEGG function enrichment analyses, and the regulation of T cell differentiation in thymus, and positive regulation of T cell differentiation in thymus were enriched in COAD, strongly suggesting MCU complex-mediated mitochondrial calcium homeostasis was closely correlated with the function of T cells. CD8^+^/CD4^+^ T cells within the TME were exposed to various signals that ultimately determined functional outcomes. Consistent with the function enrichment results, the compositions of Tregs and M0/M2 macrophages were significantly increased in MCUrisk-high group, whereas CD4^+^ memory T cells was significantly decreased in MCUrisk-high group. Consistent with the above results, the immune score was also observed to be negatively correlated with the risk score, suggesting both the decreased infiltration of immune cells with tumor cytotoxicity and the increased infiltration of pro-tumor immune cells contributed to the formation of tumor immnuesuppressive microenvironment in MCUrisk-high group in COAD. Singh *et al.* demonstrated that an elevation in mitochondrial Ca^2+^ levels involved into the C12-induced apoptosis in neutrophils 39. Based on the present study and previous studies, we inferred that the tumor immnuesuppressive microenvironment might contribute the poor prognosis in MCUrisk-high group in COAD.

Targeting cancer cells by modulating the immune response has become an important new therapeutic approach in kinds of malignancies. The expressions of immune checkpoint were associated with the response to immunotherapy in kinds of malignancies. Recently, inhibition of CTLA4/B7 and PD1/PDL1 signaling has been already successfully applied to various hematologic malignancies 40. In the present study, we found that the expression levels of majority of immune checkpoints were negatively correlated with the MCUrisk, such as CD58 and CD226. Weulersse *et al.* found that CD226 was absent in a subset of dysfunctional CD8^+^ T cells present in peripheral blood of healthy individuals, strongly suggesting CD226 was associated with the function of CD8^+^ T cells 41. Consist with the above study, the expression of CD226 was significantly negatively correlated with MCUrisk, which indicated that low expression of CD226-mediated dysfunction of CD8^+^ Tcells might contribute to the formation of tumor immunosuppressive microenvironment in COAD patient with high-MCUrisk. CD155-CD226 checkpoints functioned key roles in cancer cell/CD8^+^ T cell interaction. Feng *et al.* also reported that BCL9 regulated CD226 checkpoints to promote CD8^+^ T cell tumor infiltration in mouse colon cancer models 42. Moreover, Demetriou *et al.* reported that the CD2-CD58 system countered T cells exhaustion 43. Shen reported that loss of CD58 led to decreased T cell-mediated cytotoxicity, T-cell activation and antitumor efficacy 44. Romain *et al.* reported that CD2 on T cells was associated with directional migration and that the interaction between CD2 on T cells and CD58 on lymphoma cells accelerated killing and serial killing, and the elevated CD58 expression on pretreatment tumor samples in patients with relapsed or refractory large B cell lymphomas treated with CD19-specific CAR T cell therapy was associated with complete clinical response and survival 45. Consist with the above studies, the expression of CD58 was also significantly negatively correlated with MCUrisk, which indicated that low expression of CD58-mediated dysfunction of T cells might contribute to the formation of tumor immunosuppressive microenvironment in COAD patient with high-MCUrisk.

**Figure 10:**
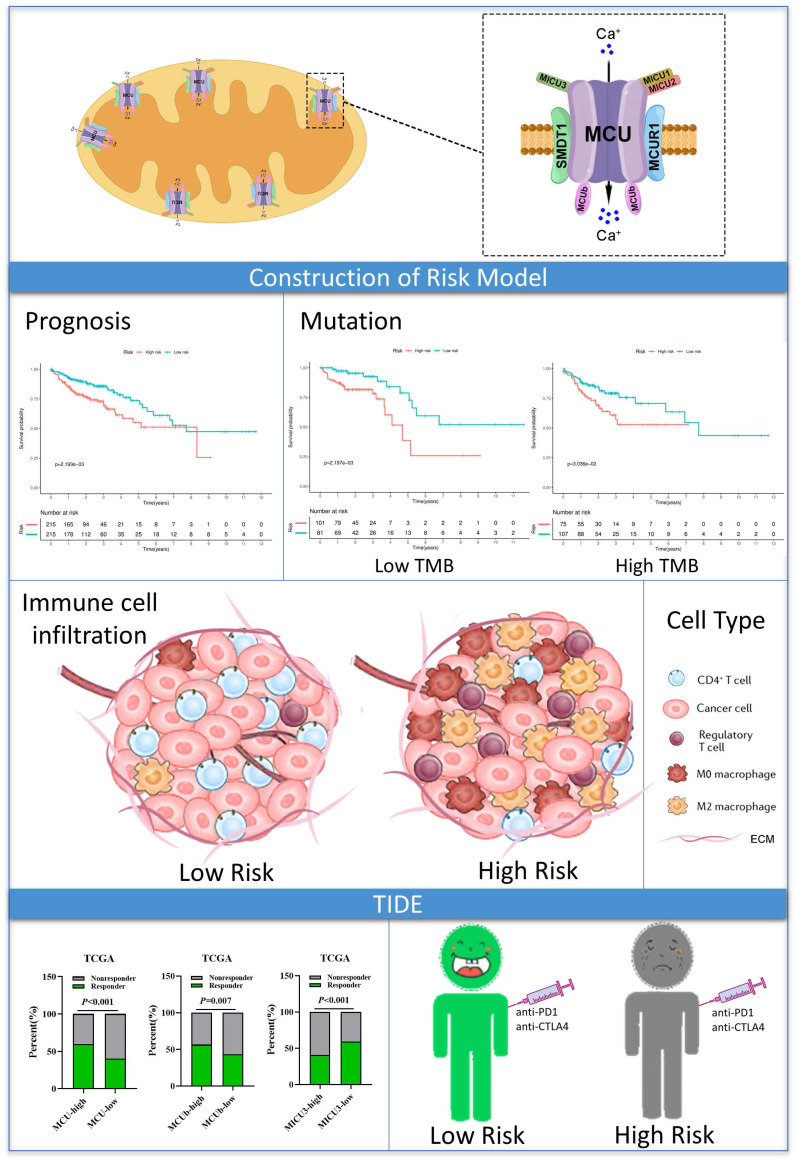
Graph summarization. The work summary graph of this study.

The advantage of our present study is that we constructed a prognostic risk model by MCU complex-associated genes that could more accurately predict OS and immunotherpay response in COAD (**Figure 10**). However, there are some limitations in the present study. First, the mRNA expression of the MCU complex members should be validated in more COAD samples. Second, our present study backs evidence that TP53 mutation is closely associated with mitochondrial calcium dyehomeostasis, although exactly how this happens remains unclear. Third, the exact mechanism underling the formation of tumor immunosuppressive microenvironment in COAD patients with high MCUrisk is still unclear. Fourth, the causal relationship between the abnormal infiltration of immune cells and the expressions of MCU complex members in COAD is not well elaborated. Last, more preclinical studies and prospective clinical trials are required to reconfirm our findings.

## Conclusion

Our present study built a novel risk signature that was independently related to the overall survival of COAD. These findings provided a more accurate prediction of COAD prognosis and personalized immunetherapy for COAD patients. The mechanisms related to mitochondrial calcium uptake-associated genes and immune regulation during the initiation and development of COAD need further exploration.

## Supplementary Material

Supplementary figures and table.

## Figures and Tables

**Table 1:** The clinical information of COAD samples in TCGA and GEO datasets.

Characteristic	TCGA (n, %)	GSE39582(n, %)	GSE29623(n, %)	GSE17536(n, %)
Platform	Illumina HiSeq2000RNA sequencing platform	Affymetrix Human Genome U133 Plus 2.0 Array	Affymetrix Human Genome U133 Plus 2.0 Array	Affymetrix Human Genome U133 Plus 2.0 Array
**Samples**	**471(100%)**	**575(100.00%)**	**65(100.00%)**	**177(100.00%)**
Normal	41(8.7%)	19(3.30%)	0	0
Tumor	430(91.3%)	556(96.70%)	65(100.00%)	177(100.00%)
**Survival Status**	**430(100.00%)**	**556(100.00%)**	**65(100.00%)**	**177(100.00%)**
Death	94(21.86%)	187(33.63%)	25(38.46%)	73(41.24%)
Survival	336(78.14%)	369(66.37%)	40(61.54%)	104(58.76%)
**Age**	**430(100.00%)**	**555(99.82%)**	**NA**	**177(100.00%)**
≤66	192(44.65%)	236(42.45%)		91(51.43%)
>66	238(55.35%)	319(57.37%)		86(48.59%)
**Gender**	**430(100.00%)**	**556(100.00%)**	**65(100.00%)**	**177(100.00%)**
Female	198(46.05%)	249(44.78%)	25(38.46%)	81(45.76%)
Male	232(53.95%)	307(55.22%)	40(61.54%)	96(54.24%)
**Stage**	**419(97.44%)**	**552(99.28%)**	**NA**	**NA**
Ⅰ	73(16.98%)	32(5.76%)		
Ⅱ	165(38.37%)	258(46.40%)		
Ⅲ	121(28.14%)	203(36.51%)		
Ⅳ	60(13.95%)	59(10.61%)		
**T classification**	**429(99.77%)**	**532(95.68%)**	**65(100.00%)**	**NA**
T1	11(2.56%)	11(1.98%)	0	
T2	75(17.44%)	44(7.91%)	8(12.31%)	
T3	294(68.37%)	360(64.75%)	52(80.00%)	
T4	49(11.40%)	117(21.04%)	5(7.69%)	
**N classification**	**430(100.00%)**	**530(95.32%)**	**64(98.5%)**	**NA**
N0	253(58.84%)	295(53.05%)	32(49.23%)	
N1	100(23.26%)	131(23.56%)	25(38.46%)	
N2	77(17.90%)	98(17.63%)	7(10.77%)	
N3	0	6(1.08%)	0	
**M classification**	**378(87.90%)**	**534(96.04%)**	**64(98.5%)**	**NA**
M0	318(73.95%)	474(85.25%)	46(70.77%)	
M1	60(13.95%)	60(10.79%)	18(27.69%)	

For TNM classification, T, N, and M refer to primary tumor, regional lymph nodes, and distant metastasis, respectively. Abbreviations: TCGA, The Cancer Genome Atlas; NA, not available.

**Table 2:** The clinicopathological characteristics of COAD patients in the low-risk and high-risk groups.

Variable		High risk n=215		Low risk n=215		p-value
Age	≤66	101		91		0.332
	> 66	114		124		
Gender	Female	95		103		0.439
	Male	120		112		
Stage	I-II	100		138		**0.000**
	III-IV	108		73		
	N/A	7		4		
T	T1/2	39		47		0.323
	T3/4	176		167		
	N/A			1		
N	N0	111		142		**0.002**
	N1/N2	104		73		
M	M0	156		162		**0.042**
	M1	38		22		
	MX	21		31		
Survival status	Alive	158		178		**0.020**
	Dead	57		37		
						

## Abbreviations

COAD: colon adenocarcinoma
MCU: mitochondrial calcium upiporter
DEGs: differentially expressed genes
TCGA: The Cancer Genome Atlas
GEO: Gene Expression Omnibus
PPI: protein-protein Interaction
ROC: receiver operating characteristic curve
OS: overall survival
DFS: disease-free survival
LASSO: least absolute shrinkage and selection operator
GEPIA: gene expression profiling interactive analysis
GO: gene ontology
TMB: tumor mutation burden
TME: tumor microenvironment
