# Supplementary material for: Identification and Validation of a Mitochondria Calcium Uptake-Related Gene Signature for Predicting Prognosis in COAD
Source: J Cancer. 2023 Mar 21;14(5):741–58. doi: 10.7150/jca.81811 (PMC10088886; doi:10.7150/jca.81811)
Supplement: Supplementary file 1 — Supplementary figures and table. [file jcav14p0741s1.pdf]

Identification and Validation of a Mitochondria Calcium Uptake-Related Gene Signature for Predicting Prognosis in COAD

Jianjun Zhu[1#], Wentao Zhang[2#], Jingjia Chang[2], Jin Wu[3], Hao Wu[2], Xintong Zhang[2], Zhigao Ou[2], Ting Tang[2], Li Li[2], Ming Liu[2*], Yongfan Xin[4*]

[1]Department of Cell Biology and Medical Genetics, Shanxi Medical University, Department of Hepatological Surgery, First Hospital of Shanxi Medical University, Taiyuan, Shanxi, China.
[2]Department of Cell Biology and Medical Genetics, Shanxi Medical University, Taiyuan, Shanxi, China.
[3]Department of Molecular & Cellular Biology, Roswell Park Comprehensive Cancer Center, Elm and Carlton Streets, Buffalo, NY 14263, USA.
[4]Department of oncology and vascular intervention, First Hospital of Shanxi Medical University, Taiyuan, Shanxi, China.

#Jianjun Zhu and Wentao Zhang contributed equally to the work.

Corresponding authors: **Yongfan Xin,** E-mail:15541190713@163.com; Department of oncology and vascular intervention, First Hospital of Shanxi Medical University, Taiyuan, Shanxi, China. **Ming Liu**, E-mail: lium0421@163.com; Department of Cell Biology and Medical Genetics, Shanxi Medical University,Taiyuan, Shanxi, China.

Supplementary figure legend

Figure S1 The mRNA expressions of MCU complex members in colon cancer (GSE39582). $*P < 0.05$; $**P < 0.01$.

Figure S2 The relationships between the mRNA expression of MCU complex members and clinicopathological characteristics

Figure S3 The prognostic value of individual MCU complex members in COAD patients (GEPIA). **A**, OS. **B**, RFS

Figure S4 Genetic variations of the mutation of MCU complex members (cBioPortal), and Gene-gene interaction network, protein-protein interaction network and correlation analysis of the MCU complex members in colon cancer. **A**, Overview the genetic variations analyses of MCU complex members. **B**, Correlation analysis between the mRNA expression of MCU complex members and tumor mutational burden (TMB) of COAD patients. C, Co-mutation analysis between the MCU complex members and high-frequency genes in COAD. D, Protein-protein interaction network analysis through String database. E, Correlation analysis of the MCU complex members. F, Gene-gene interaction network analysis of MCU complex members through GeneMANIA.

Figure S5 The relationships between the mRNA expression of MCU complex members and immune cell infiltration.

Figure S6 The relationships between the mRNA expression of MCU complex members and immune checkpoint and immunotherapy. **a**, The expression of PD1, PDL-L1 and CTLA4 in the low-expression and high-expression groups based on the median expressions of each MCU complex members in COAD, respectively. **b**, The distribution of responder and non-responder to anti CTLA-4/PD-1 immunotherapy in the low-expression and high-expression groups based on the median expressions of each MCU complex members in COAD, respectively.

Figure S7 Correlation analysis between the expression of MCU complex members and immune checkpoint molecules.

Figure S8 The relationships between risk scores and clinicopathological characteristics of COAD patients from training set.

Figure S9 Validation of the risk signature in the validation datasets (GSE29623 and GSE39582). **A,** Heatmap of mRNA expression of MCU complex members in low-risk and high-risk group. **B,** The mRNA expression of cMCU complex members in low-risk and high-risk group. **C,** K-M survival between low-risk and high-risk group. **D,** ROC curve and AUC at 1, 3, and 5 years for the risk score. ***P* < 0.01; ****P* < 0.001.

Figure S10 Functional enrichment analysis of MCU and its co-expression genes. Gene Ontology (GO) analysis.

Figure S11 The associations between risk scores and immune cell infiltration. **A**, The abundance of 22 immune infiltrates between the low-risk and high-risk group. **B**, The relationship between risk score and the estimate score.C, The relationship between risk score and the expression of macrophages marker genes.

Figure S12 The associations between risk scores and immune cell infiltration.

Supplementary Table

Supplementary Table 1 Detail information of MCU complex members.

Gene symbol	Full name	Function of the encoded protein	Also known as
MCU	mitochondrial calcium uniporter	This gene encodes a calcium transporter that localizes to the mitochondrial inner membrane. The encoded protein interacts with mitochondrial calcium uptake 1.	HsMCU; C10orf42; CCDC109A
MCUb	mitochondrial calcium uniporter dominant negative subunit beta	Predicted to enable calcium channel inhibitor activity. Predicted to be involved in calcium import into the mitochondrion and mitochondrial calcium ion homeostasis. Located in mitochondrion and nucleoplasm. Is integral component of mitochondrial inner membrane. Part of uniplex complex	CCDC109B
MCUR1	mitochondrial calcium uniporter regulator 1	Involved in calcium import into the mitochondrion and positive regulation of mitochondrial calcium ion concentration. Is integral component of mitochondrial inner membrane	FMP32; C6orf79; CCDC90A
SMDT1	single-pass membrane protein with aspartate rich tail 1	This gene encodes a core regulatory component of a calcium channel in the mitochondrial inner membrane	DDDD; EMRE; C22orf32
MICU1	mitochondrial calcium uptake 1	This gene encodes an essential regulator of mitochondrial Ca2+ uptake under basal conditions. The encoded protein interacts with the mitochondrial calcium uniporter, a mitochondrial inner membrane Ca2+ channel, and is essential in preventing mitochondrial Ca2+ overload, which can cause excessive production of reactive oxygen species and cell stress.	CALC; EFHA3; MPXPS; CBARA1; ara CALC
MICU2	mitochondrial calcium uptake 2	Enables protein heterodimerization activity. Involved in calcium import into the mitochondrion and negative regulation of mitochondrial calcium ion concentration. Located in mitochondrial inner membrane and mitochondrial intermembrane space. Part of uniplex complex	EFHA1; 1110008L20Rik
MICU3	mitochondrial calcium uptake family member 3	Predicted to enable calcium ion binding activity. Predicted to be involved in calcium import into the mitochondrion and mitochondrial calcium ion homeostasis. Predicted to be located in mitochondrial inner membrane. Predicted to be part of uniplex complex	EFHA2

Figure S1

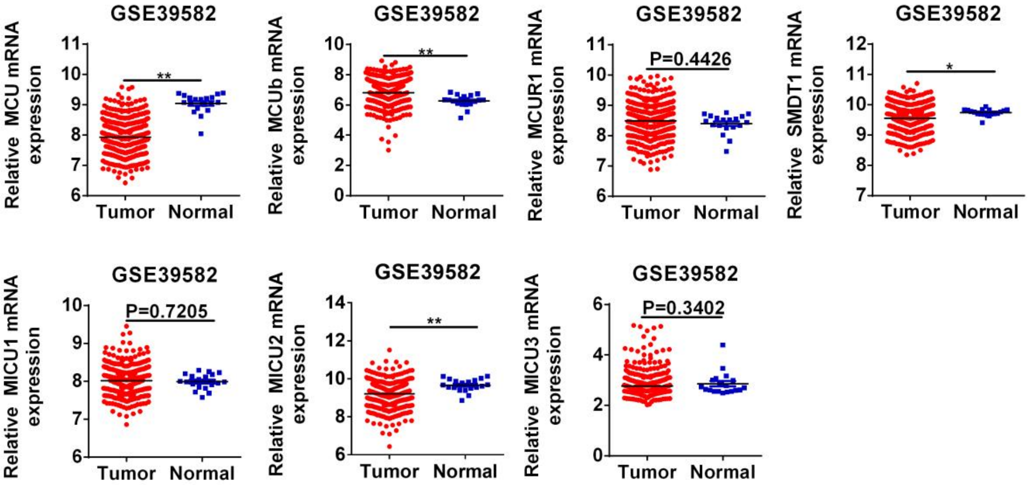
GSE39582
Relative MCU mRNA expression
**
Tumor
Normal
GSE39582
Relative MCUb mRNA expression
**
Tumor
Normal
GSE39582
Relative MCUR1 mRNA expression
P=0.4426
Tumor
Normal
GSE39582
Relative SMDT1 mRNA expression
*
Tumor
Normal
GSE39582
Relative MICU1 mRNA expression
P=0.7205
Tumor
Normal
GSE39582
Relative MICU2 mRNA expression
**
Tumor
Normal
GSE39582
Relative MICU3 mRNA expression
P=0.3402
Tumor
Normal

Figure S2

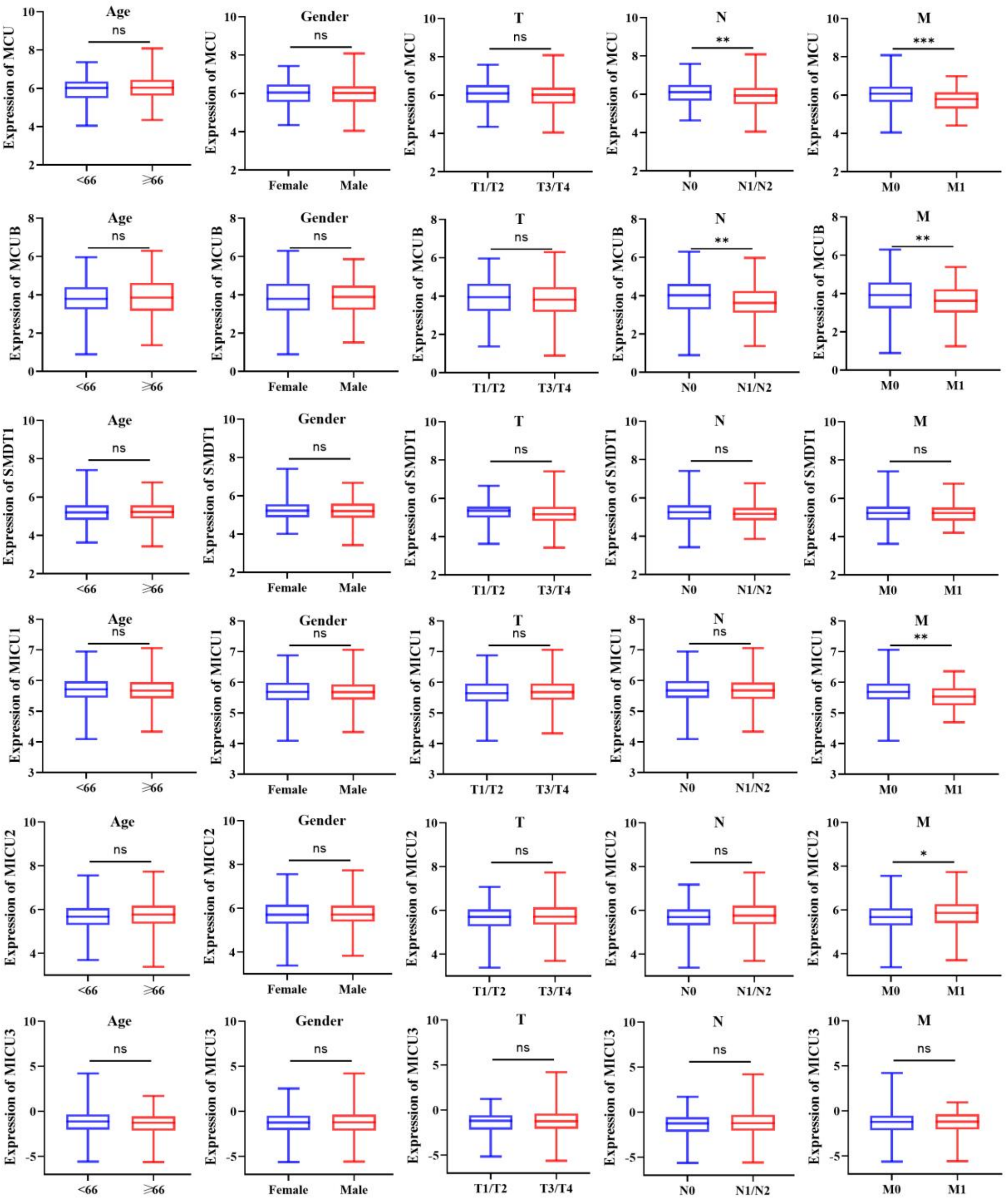

Expression of MCU
Age
ns
<66
⩾66
Gender
ns
Female
Male
T
ns
T1/T2
T3/T4
N
**
N0
N1/N2
M
***
M0
M1
Expression of MCUB
Age
ns
Gender
ns
T
ns
N
**
M
**
Expression of SMDT1
Age
ns
Gender
ns
T
ns
N
ns
M
ns
Expression of MICU1
Age
ns
Gender
ns
T
ns
N
ns
M
**
Expression of MICU2
Age
ns
Gender
ns
T
ns
N
ns
M
*
Expression of MICU3
Age
ns
Gender
ns
T
ns
N
ns
M
ns

Figure S3

A

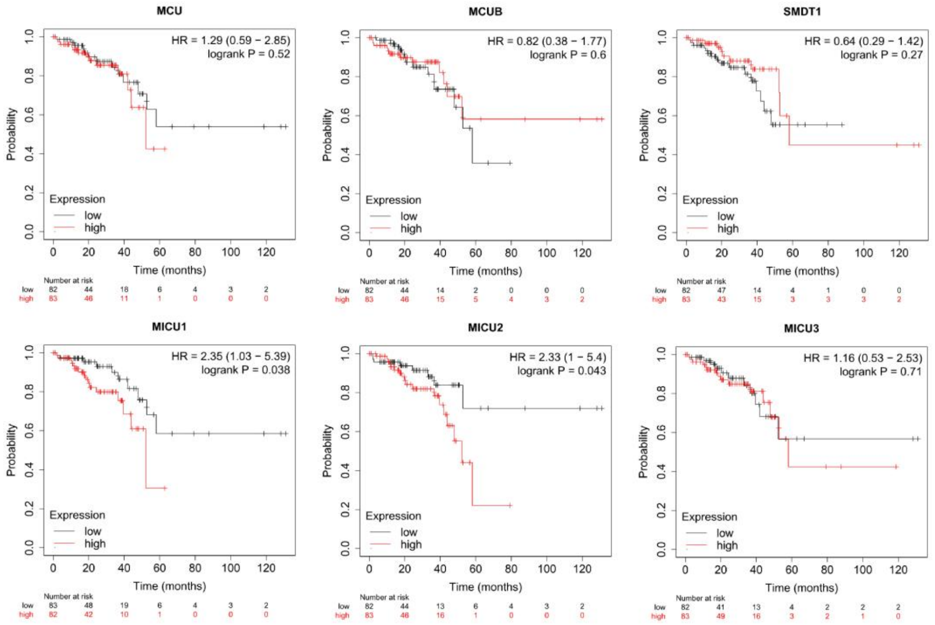
MCU
HR = 1.29 (0.59 − 2.85)
logrank P = 0.52
MCUB
HR = 0.82 (0.38 − 1.77)
logrank P = 0.6
SMDT1
HR = 0.64 (0.29 − 1.42)
logrank P = 0.27
MICU1
HR = 2.35 (1.03 − 5.39)
logrank P = 0.038
MICU2
HR = 2.33 (1 − 5.4)
logrank P = 0.043
MICU3
HR = 1.16 (0.53 − 2.53)
logrank P = 0.71
Expression
low
high
Probability
Time (months)
Number at risk

B

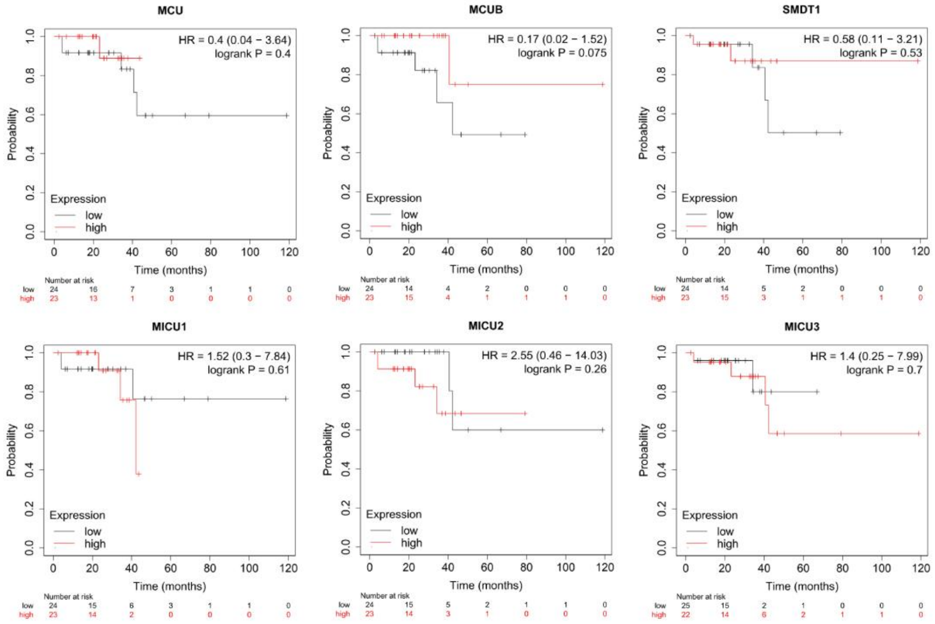
MCU
HR = 0.4 (0.04 − 3.64)
logrank P = 0.4
MCUB
HR = 0.17 (0.02 − 1.52)
logrank P = 0.075
SMDT1
HR = 0.58 (0.11 − 3.21)
logrank P = 0.53
MICU1
HR = 1.52 (0.3 − 7.84)
logrank P = 0.61
MICU2
HR = 2.55 (0.46 − 14.03)
logrank P = 0.26
MICU3
HR = 1.4 (0.25 − 7.99)
logrank P = 0.7
Expression
low
high
Probability
Time (months)
Number at risk

Figure S4

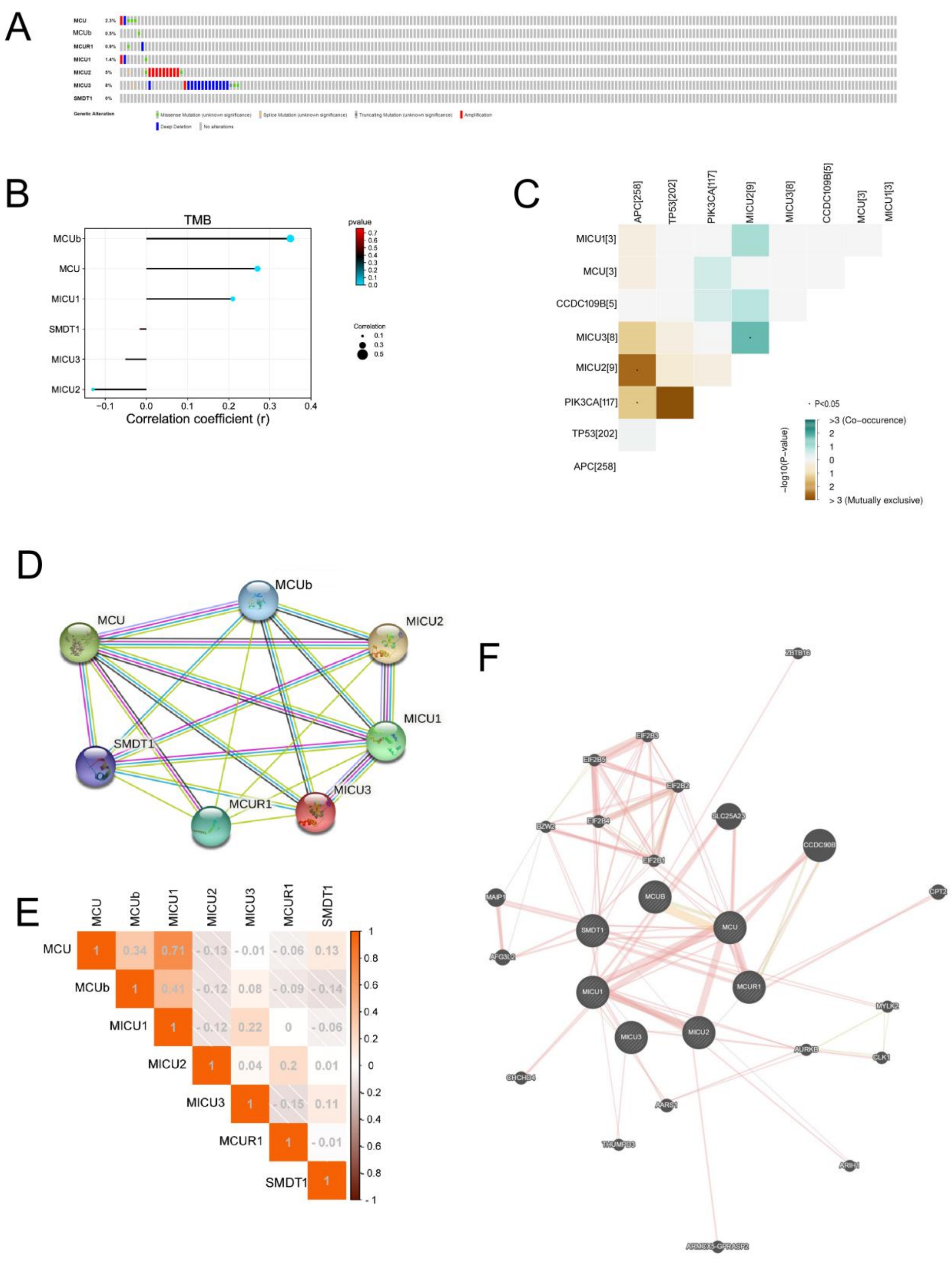

A
MCU
MCUb
MCUR1
MICU1
MICU2
MICU3
SMDT1
Genetic Alteration
B
TMB
MCUb
MCU
MICU1
SMDT1
MICU3
MICU2
−0.1
0.0
0.1
0.2
0.3
0.4
Correlation coefficient (r)
pvalue
0.7
0.6
0.5
0.4
0.3
0.2
0.1
0.0
Correlation
0.1
0.3
0.5
C
APC[258]
TP53[202]
PIK3CA[117]
MICU2[9]
MICU3[8]
CCDC109B[5]
MCU[3]
MICU1[3]
P<0.05
>3 (Co−occurence)
2
1
0
1
2
> 3 (Mutually exclusive)
−log10(P−value)
D
MCUb
MCU
MICU2
MICU1
SMDT1
MICU3
MCUR1
E
MCU
MCUb
MICU1
MICU2
MICU3
MCUR1
SMDT1
1
0.34
0.71
- 0.13
- 0.01
- 0.06
0.13
0.41
- 0.12
0.08
- 0.09
- 0.14
- 0.12
0.22
0
- 0.06
0.04
0.2
0.01
- 0.15
0.11
- 0.01
0.8
0.6
0.4
0.2
- 0.2
- 0.4
- 0.6
- 0.8
- 1
F
ZBTB16
EIF2B3
EIF2B5
EIF2B2
BZW2
EIF2B4
SLC25A23
CCDC90B
EIF2B1
MAIP1
MCUB
CPT2
SMDT1
MCU
AFG3L2
MICU1
MCUR1
MYLK2
MICU3
MICU2
AURKB
CLK1
CHCHD4
AARS1
THUMPD3
ARIH1
ARMCX5-GPRASP2

Figure S5

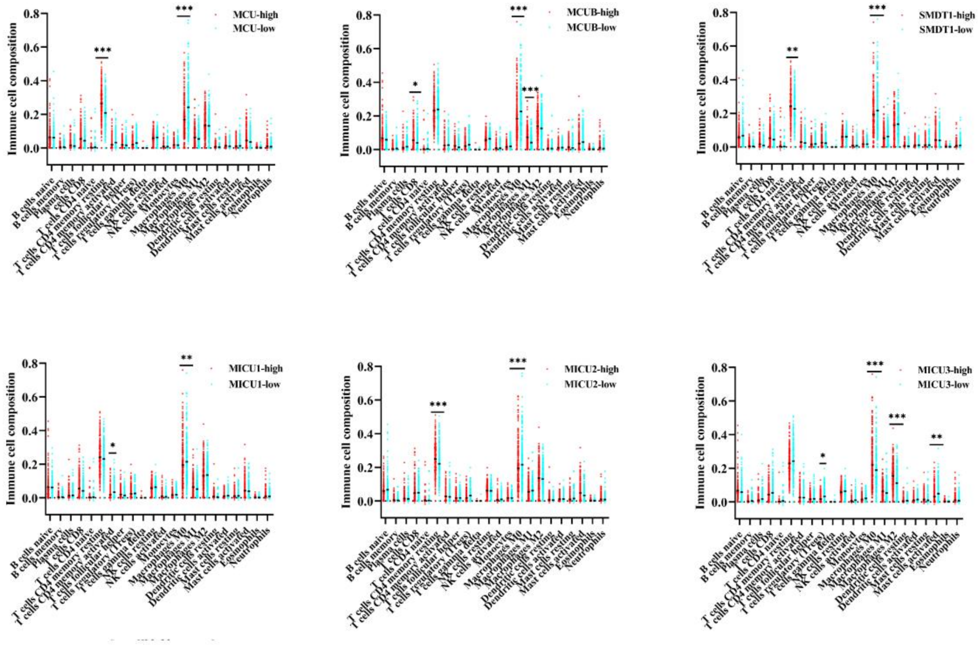
Immune cell composition
MCU-high
MCU-low
MCUB-high
MCUB-low
SMDT1-high
SMDT1-low
MICU1-high
MICU1-low
MICU2-high
MICU2-low
MICU3-high
MICU3-low

Figure S6

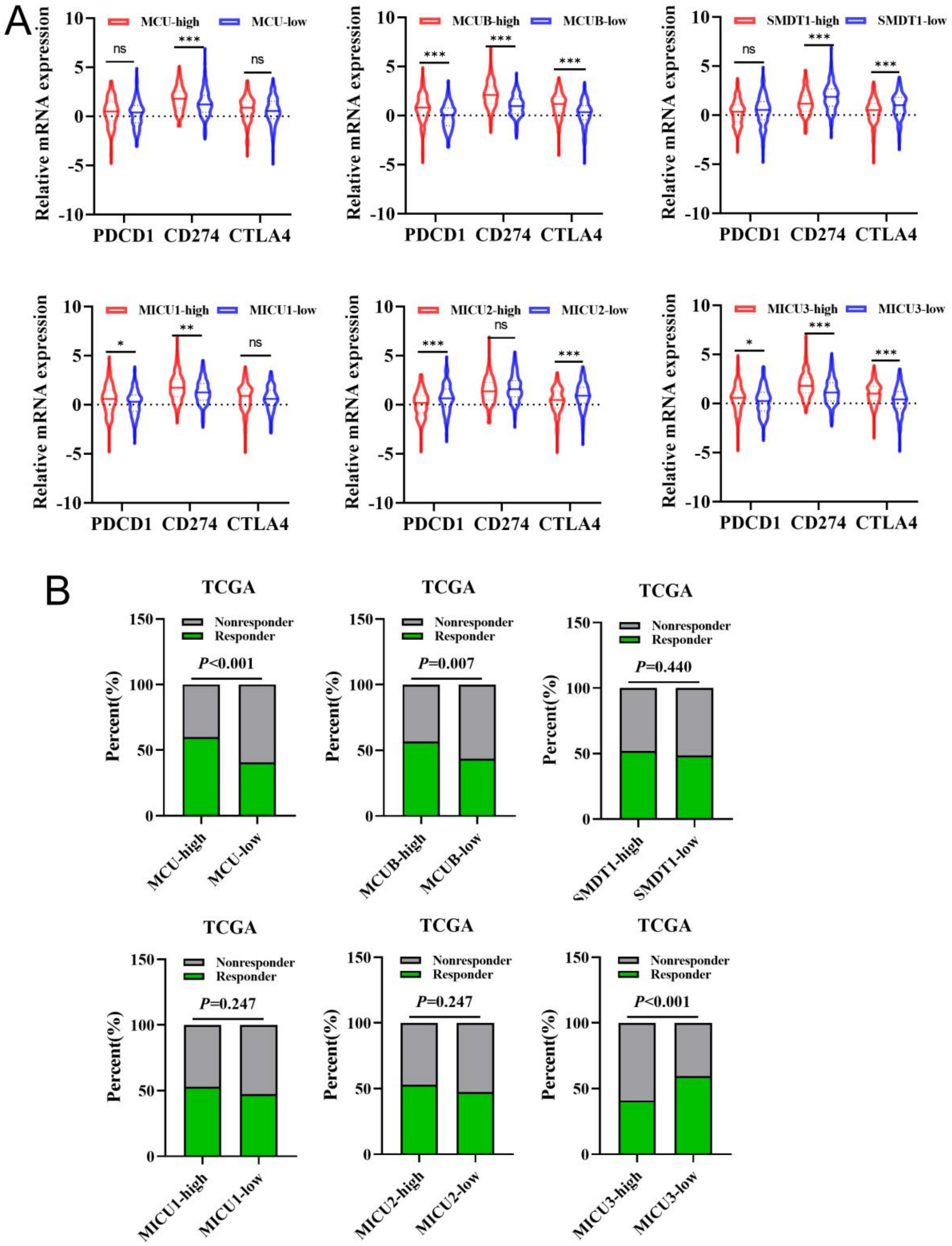
A
MCU-high
MCU-low
MCUB-high
MCUB-low
SMDT1-high
SMDT1-low
MICU1-high
MICU1-low
MICU2-high
MICU2-low
MICU3-high
MICU3-low
Relative mRNA expression
PDCD1
CD274
CTLA4
ns
***
**
*
B
TCGA
Nonresponder
Responder
Percent(%)
P<0.001
P=0.007
P=0.440
P=0.247
P=0.247
P<0.001
MCU-high
MCU-low
MCUB-high
MCUB-low
SMDT1-high
SMDT1-low
MICU1-high
MICU1-low
MICU2-high
MICU2-low
MICU3-high
MICU3-low

Figure S7

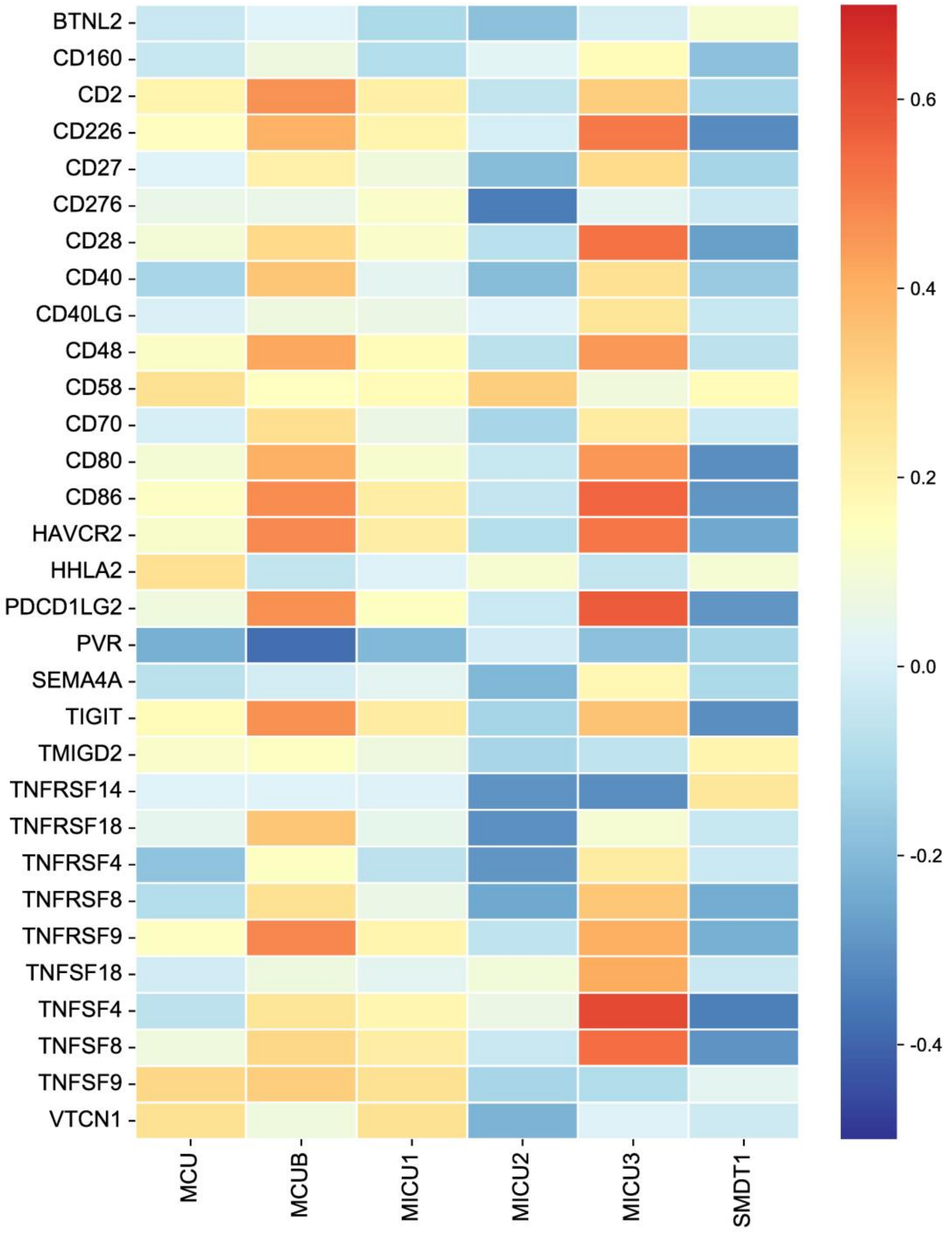
BTNL2
CD160
CD2
CD226
CD27
CD276
CD28
CD40
CD40LG
CD48
CD58
CD70
CD80
CD86
HAVCR2
HHLA2
PDCD1LG2
PVR
SEMA4A
TIGIT
TMIGD2
TNFRSF14
TNFRSF18
TNFRSF4
TNFRSF8
TNFRSF9
TNFSF18
TNFSF4
TNFSF8
TNFSF9
VTCN1
MCU
MCUB
MICU1
MICU2
MICU3
SMDT1
0.6
0.4
0.2
0.0
-0.2
-0.4

Figure S8

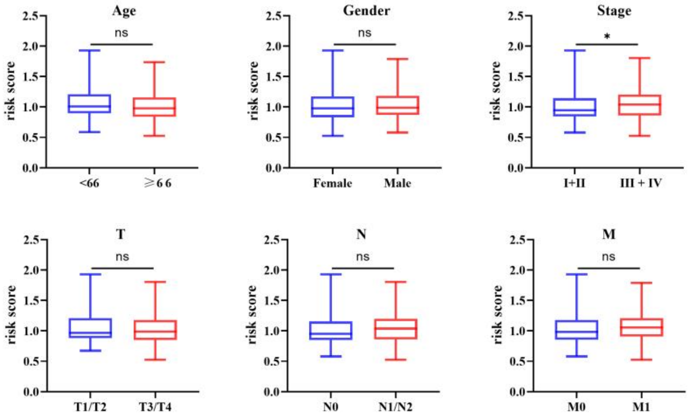
Age
ns
risk score
<66
≥66
Gender
ns
Female
Male
Stage
*
I+II
III + IV
T
ns
T1/T2
T3/T4
N
ns
N0
N1/N2
M
ns
M0
M1

Figure S9

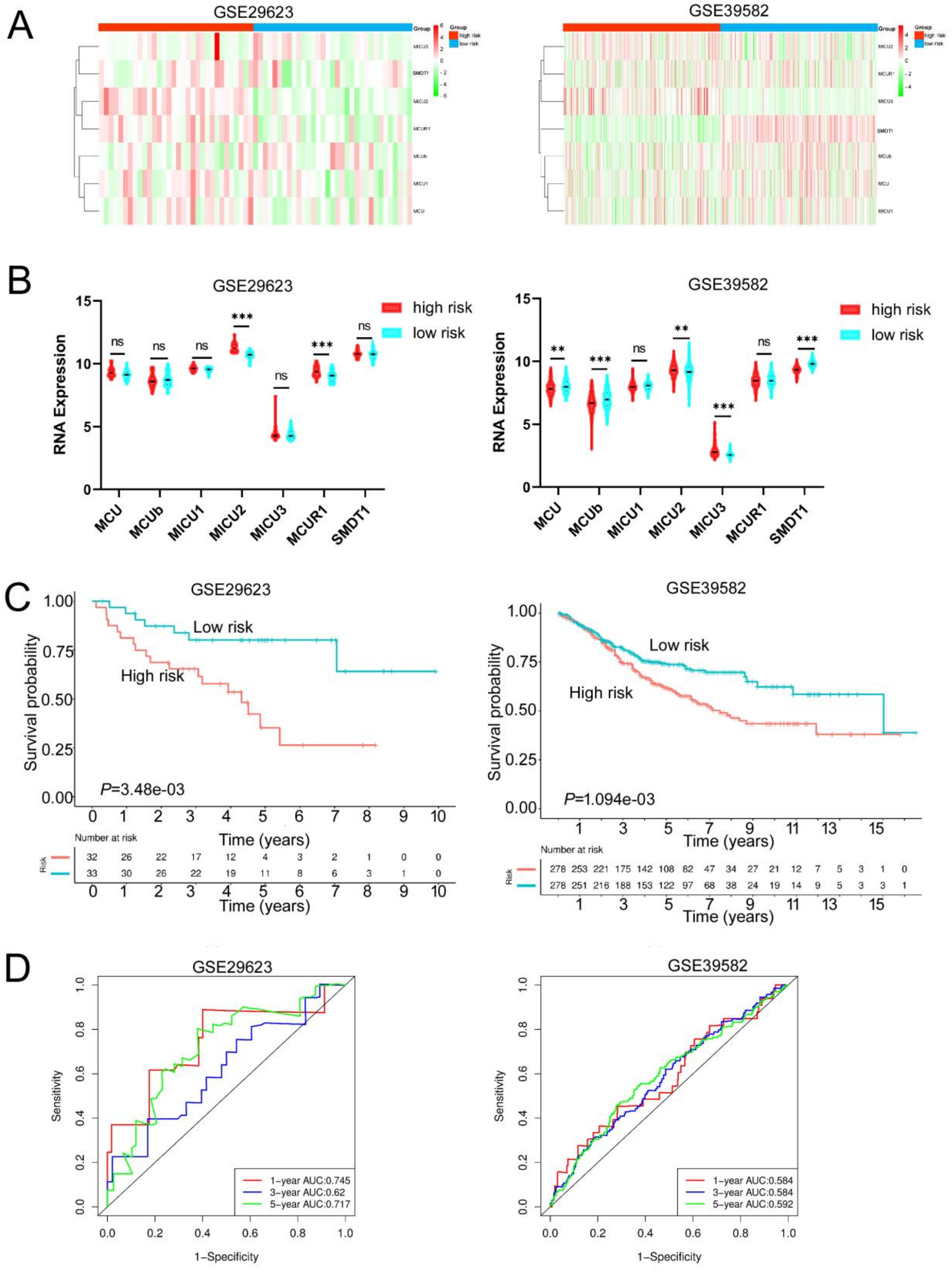

A
GSE29623
GSE39582
Group
high risk
low risk
B
GSE29623
GSE39582
RNA Expression
high risk
low risk
MCU
MCUb
MICU1
MICU2
MICU3
MCUR1
SMDT1
C
GSE29623
Low risk
High risk
Survival probability
P=3.48e-03
Time (years)
Number at risk
GSE39582
P=1.094e-03
D
GSE29623
Sensitivity
1−Specificity
1−year AUC:0.745
3−year AUC:0.62
5−year AUC:0.717
GSE39582
1−year AUC:0.584
3−year AUC:0.584
5−year AUC:0.592

Figure S10

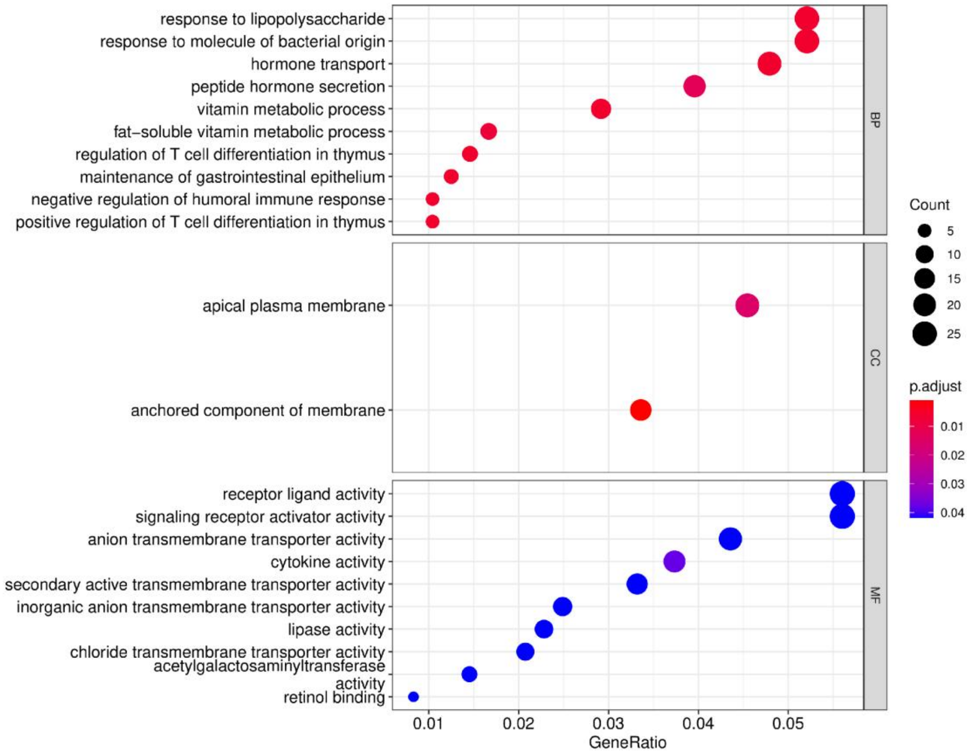

response to lipopolysaccharide
response to molecule of bacterial origin
hormone transport
peptide hormone secretion
vitamin metabolic process
fat−soluble vitamin metabolic process
regulation of T cell differentiation in thymus
maintenance of gastrointestinal epithelium
negative regulation of humoral immune response
positive regulation of T cell differentiation in thymus
BP
apical plasma membrane
anchored component of membrane
CC
receptor ligand activity
signaling receptor activator activity
anion transmembrane transporter activity
cytokine activity
secondary active transmembrane transporter activity
inorganic anion transmembrane transporter activity
lipase activity
chloride transmembrane transporter activity
acetylgalactosaminyltransferase activity
retinol binding
MF
0.01
0.02
0.03
0.04
0.05
GeneRatio
Count
5
10
15
20
25
p.adjust
0.01
0.02
0.03
0.04

Figure S11

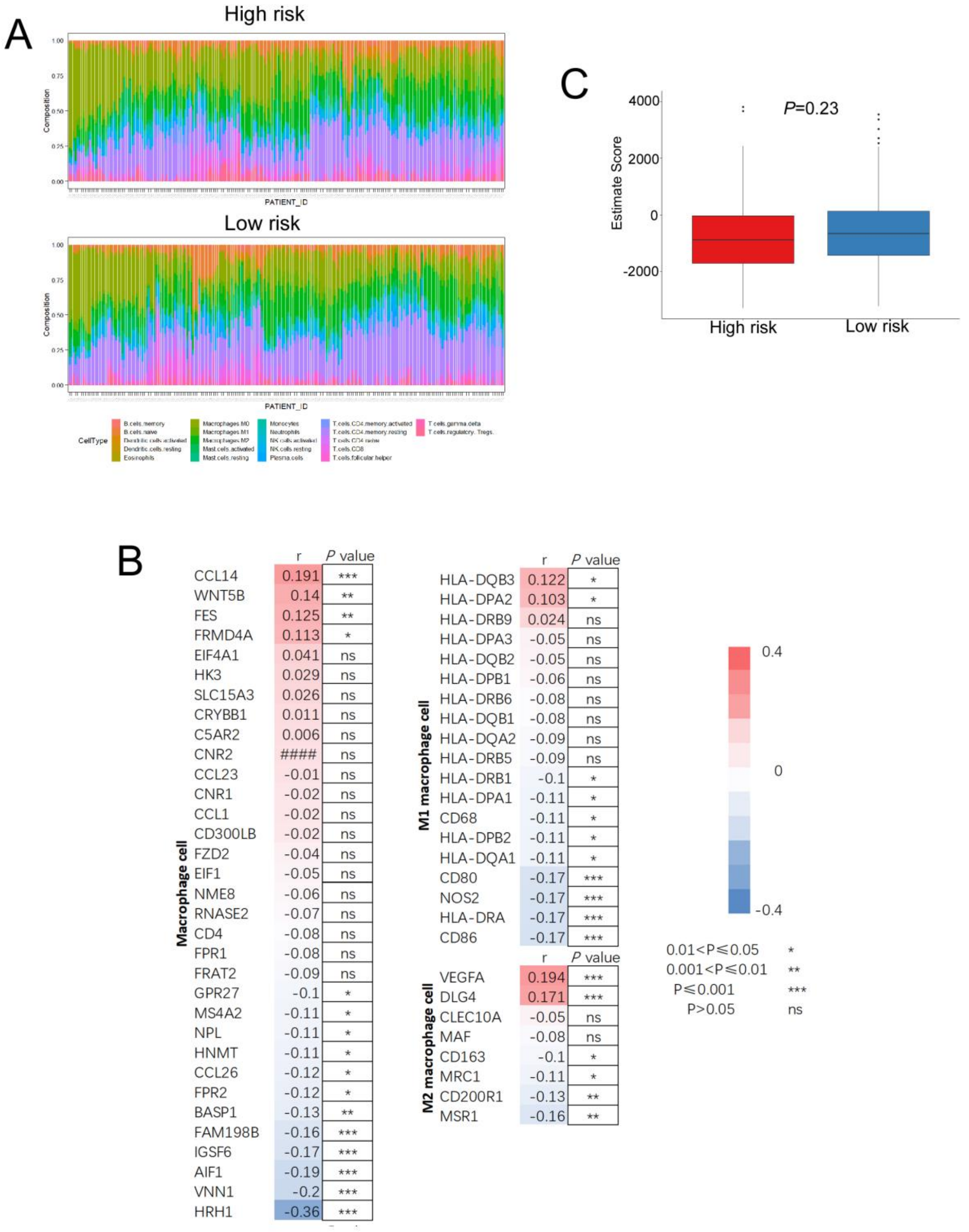
A
High risk
Composition
PATIENT_ID
Low risk
Composition
PATIENT_ID
CellType
C
P=0.23
4000
2000
0
-2000
Estimate Score
High risk
Low risk
B
Macrophage cell
r
P value
CCL14 0.191 ***
WNT5B 0.14 **
FES 0.125 **
FRMD4A 0.113 *
EIF4A1 0.041 ns
HK3 0.029 ns
SLC15A3 0.026 ns
CRYBB1 0.011 ns
C5AR2 0.006 ns
CNR2 #### ns
CCL23 -0.01 ns
CNR1 -0.02 ns
CCL1 -0.02 ns
CD300LB -0.02 ns
FZD2 -0.04 ns
EIF1 -0.05 ns
NME8 -0.06 ns
RNASE2 -0.07 ns
CD4 -0.08 ns
FPR1 -0.08 ns
FRAT2 -0.09 ns
GPR27 -0.1 *
MS4A2 -0.11 *
NPL -0.11 *
HNMT -0.11 *
CCL26 -0.12 *
FPR2 -0.12 *
BASP1 -0.13 **
FAM198B -0.16 ***
IGSF6 -0.17 ***
AIF1 -0.19 ***
VNN1 -0.2 ***
HRH1 -0.36 ***
M1 macrophage cell
r
P value
HLA-DQB3 0.122 *
HLA-DPA2 0.103 *
HLA-DRB9 0.024 ns
HLA-DPA3 -0.05 ns
HLA-DQB2 -0.05 ns
HLA-DPB1 -0.06 ns
HLA-DRB6 -0.08 ns
HLA-DQB1 -0.08 ns
HLA-DQA2 -0.09 ns
HLA-DRB5 -0.09 ns
HLA-DRB1 -0.1 *
HLA-DPA1 -0.11 *
CD68 -0.11 *
HLA-DPB2 -0.11 *
HLA-DQA1 -0.11 *
CD80 -0.17 ***
NOS2 -0.17 ***
HLA-DRA -0.17 ***
CD86 -0.17 ***
M2 macrophage cell
r
P value
VEGFA 0.194 ***
DLG4 0.171 ***
CLEC10A -0.05 ns
MAF -0.08 ns
CD163 -0.1 *
MRC1 -0.11 *
CD200R1 -0.13 **
MSR1 -0.16 **
0.4
0
-0.4
0.01<P≤0.05 *
0.001<P≤0.01 **
P≤0.001 ***
P>0.05 ns

Figure S12

Activated CD4 T cell

Gene	r	*P* value
DUSP2	0.056	ns
KNTC1	-0.03	ns
CCR7	-0.03	ns
PSAT1	-0.03	ns
IARS	-0.05	ns
PRC1	-0.13	ns
CCL5	-0.13	ns
CCL20	-0.15	**
ETS1	-0.15	**
CCL4	-0.16	**
SELL	-0.17	***
EXO1	-0.17	***
AIM2	-0.18	***
NUF2	-0.19	***
CCNB1	-0.19	***
ITK	-0.2	***
BRIP1	-0.22	***
TRAT1	-0.23	***
SAMSN1	-0.26	***
KIF11	-0.28	***
BIRC3	-0.29	***
RTKN2	-0.3	***
RGS1	-0.31	***
ESCO2	-0.38	***
EXOC6	-0.43	***

Central memory CD4 T cell

Gene	r	*P* value
ELMO2	0.331	***
GSS	0.301	***
ATHL1	0.23	***
STX4	0.217	***
TRADD	0.083	ns
COL4A1	0.04	ns
IFITM2	0.022	ns
VIM	-0.02	ns
ITGB2	-0.02	ns
BZW2	-0.03	ns
NDUFB9	-0.04	ns
LSP1	-0.04	ns
SFXN3	-0.05	ns
GLIPR1	-0.08	ns
FYN	-0.09	ns
SIRPG	-0.11	*
CYLD	-0.16	***
XRCC6	-0.18	***
AQP3	-0.21	***
ANXA2P2	-0.22	***
AHNAK	-0.23	***
CD63	-0.24	***
BMI1	-0.26	***
ITGB1	-0.28	***
SMAD4	-0.29	***
KLF5	-0.31	***
ABHD3	-0.42	***

Effector memory CD4 T cell

Gene	r	*P* value
IL34	0.315	***
REPS1	0.167	***
TAL1	0.098	*
CASQ1	0.07	ns
EZH2	0.008	ns
PTGS1	-0	ns
NEFL	-0.01	ns
DOCK9	-0.02	ns
USP9Y	-0.02	ns
ATM	-0.02	ns
DARS	-0.03	ns
CD300E	-0.04	ns
SCG2	-0.06	ns
SIGLEC14	-0.06	ns
SIGLEC6	-0.07	ns
SDPR	-0.09	ns
WIPF1	-0.12	*
PDGFRL	-0.14	**
TIPIN	-0.18	***
UQCRB	-0.19	***
GDE1	-0.2	***
TFEC	-0.2	***
TPK1	-0.23	***
ZCRB1	-0.26	***
EXOSC9	-0.26	***
CASP3	-0.41	***
NCOA4	-0.48	***

T follicular helper cell

Gene	r	*P* value
CHST12	0.335	***
CDK5R1	0.327	***
RAE1	0.27	***
SYNM	0.168	***
CEBPA	0.166	***
TYRO3	0.107	*
CD83	0.097	*
LRRC32	0.076	ns
MC5R	0.058	ns
B3GAT1	0.048	ns
DPP4	0.042	ns
CSF1R	#####	ns
NCR2	-0.01	ns
NCAM1	-0.01	ns
CECR1	-0.01	ns
BCL6	-0.01	ns
PDCD1	-0.01	ns
MICA	-0.02	ns
SIGLEC9	-0.02	ns
PDCD6	-0.03	ns
SIGLEC7	-0.05	ns
CLEC10A	-0.05	ns
NRP1	-0.1	*
CLIC3	-0.11	*
PRDX1	-0.13	**
CD84	-0.13	**
GPR18	-0.13	**
CD200	-0.14	**
PDCD1LG2	-0.15	**
CLEC4A	-0.19	***
FGF2	-0.19	***
LGMN	-0.2	***
RAET1E	-0.25	***
IVNS1ABP	-0.3	***
CTSS	-0.38	***

T cells regulator

Gene	r	*P* value
L1CAM	0.297	***
LRP1	0.218	***
PTGIR	0.189	***
PRSS23	0.124	*
STAB1	0.118	*
FOXP3	0.051	ns
CD72	0.032	ns
MARCO	-0.01	ns
LRRC42	-0.11	*
PELO	-0.11	*
MRC1	-0.11	*
CLEC5A	-0.14	**
MS4A6A	-0.16	**
ITGA4	-0.17	***
PLEK	-0.17	***
MMP12	-0.18	***
ST8SIA4	-0.19	***
MNDA	-0.2	***
LIPA	-0.29	***

Type 1 T helper cell

Gene	r	*P* value
IGF2	0.228	***
TRAF1	0.228	***
HUNK	0.203	***
RYR1	0.201	***
DUSP14	0.169	***
CD151	0.141	**
MAN1B1	0.134	**
LTC4S	0.125	**
FURIN	0.111	*
P2RX5	0.075	ns
DAB1	0.063	ns
SELP	0.05	ns
SELE	0.048	ns
GFPT2	0.038	ns
CD97	0.03	ns
COL5A3	0.025	ns
METRNL	0.022	ns
DLEU7	0.016	ns
IL12A	0.015	ns
STAC	0.015	ns
GREM2	0.013	ns
COL23A1	0.001	ns
F12	-0	ns
ADAM8	-0.01	ns
CD7	-0.01	ns
COL4A4	-0.02	ns
BST1	-0.02	ns
TNFRSF1A	-0.02	ns
GPR25	-0.02	ns
HAVCR1	-0.03	ns
ICAM3	-0.03	ns
CD70	-0.04	ns
HTR2B	-0.04	ns
HSD11B1	-0.05	ns
CD6	-0.05	ns
SIT1	-0.06	ns
SKAP1	-0.06	ns
CFHR3	-0.06	ns
RGS16	-0.06	ns
TBX21	-0.06	ns
ITGB4	-0.06	ns
CHRM3	-0.07	ns
CALD1	-0.08	ns
GAB3	-0.08	ns
SIGLEC10	-0.09	ns
FCGR2C	-0.09	ns
IRF1	-0.09	ns
ZEB2	-0.1	*
RCSD1	-0.1	*
CD68	-0.11	*
CD52	-0.11	*
GATM	-0.11	*
DOC2B	-0.11	*
THUMPD2	-0.11	*
MMD	-0.11	*
ENC1	-0.13	**
CD53	-0.14	**
FBXO30	-0.14	**
TLR8	-0.15	**
CD48	-0.15	**
CLEC7A	-0.17	***
FAM134B	-0.18	***
TIGIT	-0.18	***
B3GALNT1	-0.19	***
CD96	-0.22	***
MOCOS	-0.23	***
TRAF3IP2	-0.24	***
CD47	-0.24	***
SH3KBP1	-0.25	***
EMP1	-0.26	***
SAV1	-0.27	***
SLC35B3	-0.27	***
CD59	-0.27	***
AHCYL2	-0.29	***
MDH1	-0.34	***
ALCAM	-0.35	***
BBS12	-0.36	***

Type 2 T helper cell

Gene	r	*P* value
GATA3	0.067	ns
ASB2	0.049	ns
CSRP2	0.036	ns
NRP2	-0.01	ns
DLC1	-0.04	ns
BIRC5	-0.06	ns
TMPRSS3	-0.07	ns
RBMS3	-0.07	ns
CENPF	-0.08	ns
LAMP3	-0.1	*
IL26	-0.11	*
OSBPL1A	-0.12	*
PHLDA1	-0.13	**
LAIR2	-0.13	**
CDC25C	-0.15	**
DAPK1	-0.16	**
HELLS	-0.16	***
CXCR6	-0.17	***
PLA2G4A	-0.17	***
CDC7	-0.17	***
GSTA4	-0.21	***
DUSP6	-0.22	***
PDE4B	-0.26	***
GNAI1	-0.27	***
DNAJC12	-0.3	***
EVI5	-0.31	***
RNF125	-0.32	***
DHFR	-0.33	***
RAB27B	-0.48	***

Type 17 T helper cell

Gene	r	*P* value
ABCB1	0.219	***
IL17RE	0.14	**
TNIP2	0.14	**
CD40	0.102	*
IL17F	0.057	ns
C2CD4B	0.051	ns
IL17RC	0.046	ns
ILDR1	0.032	ns
IL17RA	0.026	ns
CCR9	#####	ns
IL17A	-0.01	ns
CCDC65	-0.04	ns
IL17C	-0.04	ns
PTGDR2	-0.05	ns
ADAMTS1:	-0.05	ns
IL23A	-0.09	ns
CAMTA1	-0.09	ns
ANK1	-0.12	*
SH2D6	-0.13	**
ABCA1	-0.13	**
C2CD4A	-0.16	**
B3GALT2	-0.19	***
CEACAM3	-0.23	***
IFT80	-0.25	***
CA2	-0.26	***
LONRF3	-0.27	***
ANKRD22	-0.3	***

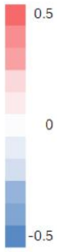

0.01<P≤0.05 *
0.001<P≤0.01 **
P≤0.001 ***
P>0.05 ns
